# Aphid gene expression following polerovirus acquisition is host species dependent

**DOI:** 10.3389/fpls.2024.1341781

**Published:** 2024-03-08

**Authors:** Sudeep Pandey, Michael Catto, Phillip Roberts, Sudeep Bag, Alana L. Jacobson, Rajagopalbabu Srinivasan

**Affiliations:** ^1^Department of Entomology, University of Georgia, Griffin, GA, United States; ^2^Department of Entomology, University of Georgia, Athens, GA, United States; ^3^Department of Entomology, University of Georgia, Tifton, GA, United States; ^4^Department of Plant Pathology, University of Georgia, Tifton, GA, United States; ^5^Department of Entomology and Plant Pathology, Auburn University, Auburn, AL, United States

**Keywords:** *Aphis gossypii*, cotton leafroll dwarf virus, acquisition, alternate hosts, vector-virus interactions

## Abstract

Upon acquisition of persistent circulative viruses such as poleroviruses, the virus particles transcytose through membrane barriers of aphids at the midgut and salivary glands via hemolymph. Such intricate interactions can influence aphid behavior and fitness and induce associated gene expression in viruliferous aphids. Differential gene expression can be evaluated by omics approaches such as transcriptomics. Previously conducted aphid transcriptome studies used only one host species as the source of virus inoculum. Viruses typically have alternate hosts. Hence, it is not clear how alternate hosts infected with the same virus isolate alter gene expression in viruliferous vectors. To address the question, this study conducted a transcriptome analysis of viruliferous aphids that acquired the virus from different host species. A polerovirus, cotton leafroll dwarf virus (CLRDV), which induced gene expression in the cotton aphid, *Aphis gossypii* Glover, was assessed using four alternate hosts, viz., cotton, hibiscus, okra, and prickly sida. Among a total of 2,942 differentially expressed genes (DEGs), 750, 310, 1,193, and 689 genes were identified in *A. gossypii* that acquired CLRDV from infected cotton, hibiscus, okra, and prickly sida, respectively, compared with non-viruliferous aphids that developed on non-infected hosts. A higher proportion of aphid genes were overexpressed than underexpressed following CLRDV acquisition from cotton, hibiscus, and prickly sida. In contrast, more aphid genes were underexpressed than overexpressed following CLRDV acquisition from okra plants. Only four common DEGs (*heat shock protein*, *juvenile hormone acid O-methyltransferase*, and two unannotated genes) were identified among viruliferous aphids from four alternate hosts. Gene ontology (GO) enrichment analysis and Kyoto Encyclopedia of Genes and Genomes (KEGG) annotations indicated that the acquisition of CLRDV induced DEGs in aphids associated with virus infection, signal transduction, immune systems, and fitness. However, these induced changes were not consistent across four alternate hosts. These data indicate that alternate hosts could differentially influence gene expression in aphids and presumably aphid behavior and fitness despite being infected with the same virus isolate.

## Introduction

Persistently transmitted single-stranded RNA phytoviruses such as poleroviruses are phloem-limited, and phloem-feeding and colonizing insects such as aphids efficiently transmit such viruses (Harris and Maramorosch, 1977; Ng and Perry, 2004). The vectors and their viruses interact intricately in these pathosystems. Ingestion of viruses occurs when aphids feed on virus-infected plants; once in the midgut, these viruses traverse into the hemocoel and then into the accessory salivary glands through transcytosis (Gray and Gildow, 2003). Also, such viruses are exclusively transmitted by specific vector species, and the specificity seems to be associated with unique receptors in vectors that mediate transcytosis (Ng and Falk, 2006; Hogenhout et al., 2008; Whitfield et al., 2015).

Persistent virus infections are known to modulate the host plant physiology and in turn alter the phenotypical traits such as leaf hue, plant growth, availability of nutrients including free amino acids and soluble carbohydrates, and profiles of volatile organic compounds (VOCs) and metabolites (Eigenbrode et al., 2002; Mauck et al., 2010). These alterations can influence vector behavior (attraction or repulsion) and performance (feeding and colonization) (Fereres and Moreno, 2009; Mauck, 2016). The host–aphid–polerovirus interactions are complex, and previous studies have reported favorable, unfavorable, and/or neutral outcomes on vector fitness (Jiménez-Martínez et al., 2004; Rajabaskar et al., 2013; Srinivasan et al., 2013; Lightle and Lee, 2014; dos Santos et al., 2016; Ghosh et al., 2016; Claudel et al., 2018; Chesnais et al., 2020, 2022; Fingu-Mabola et al., 2020; Bertasello et al., 2021; Fingu-Mabola and Francis, 2021; Jayasinghe et al., 2022). However, the majority of interactions seem to influence vector fitness and behavior positively to enhance virus transmission (Hogenhout et al., 2008).

The magnitude of the effects of virus acquisition could largely depend on virus species, vector species, host species, and their interactions. For example, the green peach aphid (*Myzus persicae* Sulzer) preferred and survived longer on potato leafroll virus (PLRV)-infected plants compared with non-infected plants (Eigenbrode et al., 2002; Srinivasan et al., 2006, 2008). On the contrary, viruliferous bird cherry-oat aphid (*Rhopalosiphum padi* L.) preferred non-infected or sham-inoculated plants compared with barley yellow dwarf virus (BYDV)-infected plants (Ingwell et al., 2012). Also, BYDV infection reduced the population growth of cereal aphids (*Sitobion avenae* F.) compared with non-infected plants (Fiebig et al., 2004). This shift or alteration in vector–virus interactions can be better understood by exploring their genetic and molecular bases (Brault et al., 2010; Li et al., 2019, 2020; Patton et al., 2021; Catto et al., 2022; Marmonier et al., 2022). For example, the underexpression of genes associated with immunity, hormone biosynthesis, and proteolytic pathways has been reported from transcriptome analysis in aphids (*S. avenae*, *Schizaphis graminum* Rondani, and *R. padi*) upon BYDV acquisition (Li et al., 2019, 2020). Similarly, the genes related to receptor activities and/or vesicular transport in *M. persicae* were underexpressed upon acquiring the turnip yellows virus (TuYV). However, the differentially expressed genes (DEGs) identified varied when the vector acquired the virus from an artificial medium compared with the virus-infected plant (Marmonier et al., 2022). Previous transcriptome studies have predominantly used only one host species as a source of virus acquisition. Only one study has been conducted to understand the discrepancies that may occur in vector fitness upon acquiring the same plant virus from different host species (Chesnais et al., 2022). Nonetheless, there remains a knowledge gap in understanding the impact of alternate hosts on virus–vector interactions as well as their fidelity across such hosts.

This study attempted to answer the above-stated question using another persistently transmitted polerovirus–aphid pathosystem. Cotton leafroll dwarf virus (CLRDV) is a phloem-limited, positive-sense, single-stranded RNA virus in the genus *Polerovirus* and belongs to the family *Solemoviridae* (Sõmera et al., 2021). The CLRDV genome is 5.8 kb long with seven open reading frames (ORFs) grouped into two blocks and separated by a non-coding region. The symptoms of CLRDV infection were first observed in cotton in the United States in Alabama in 2017, but the virus was identified in 2019 (Avelar et al., 2019). Subsequently, the virus has been reported in several cotton-producing states including Georgia (Aboughanem-Sabanadzovic et al., 2019; Tabassum et al., 2019; Alabi et al., 2020; Ali and Mokhtari, 2020; Ali et al., 2020; Faske et al., 2020; Iriarte et al., 2020; Price et al., 2020; Thiessen et al., 2020; Wang et al., 2020b). The CLRDV-infection symptoms include stunting; leaf rolling; vein yellowing; dark-green leaves; reddening of leaves, petioles, and stems; leaf puckering, crinkling, and deformation of leaf lamina; wilting; downward leaf drooping with V-shaped lamina folding; and small bolls (Cascardo et al., 2015; Silva et al., 2015; Brown et al., 2019; Sedhain et al., 2021; Pandey et al., 2022). Often, CLRDV also was detected in asymptomatic plants via reverse transcription–PCR (Bag et al., 2021). Further, CLRDV was detected in alternate hosts in the landscape in Georgia (Sedhain et al., 2021; Edula et al., 2023).

The cotton/melon aphid (*Aphis gossypii* Glover) is the only known vector of CLRDV in the United States, and it transmits the virus in a persistent and non-propagative manner (Michelotto and Busoli, 2007; Heilsnis et al., 2022, 2023). In a previous study, the aphid-mediated inoculation of CLRDV led to successful infection of hibiscus (*Hibiscus acetosella* Welw. Ex Hiern.), okra (*Abelmoschus esculentus* L.), prickly sida (*Sida spinosa* L.), Palmer amaranth (*Amaranthus palmeri* S. Wats.), and *Nicotiana benthamiana* Domin plants. Nevertheless, no CLRDV symptoms were observed in any of those host plants. Aphids were able to acquire the virus exclusively from CLRDV-infected hibiscus, okra, and prickly sida plants and subsequently inoculate the virus back to cotton plants. Although cotton, hibiscus, okra, and prickly sida belong to Malvaceae, there was a notable discrepancy in the amount of virus acquired by *A. gossypii* from those CLRDV-infected host species (Pandey et al., 2022). Also, previous studies have reported that the total fecundity and intrinsic rate of increase of *A. gossypii* varied among alternate hosts (Barman et al., 2018; Pandey et al., 2022).

This study explored how the acquisition of CLRDV from different host species affects the gene expression associated with behavior and/or fitness in its vector. Specifically, cotton, hibiscus, okra, and prickly sida were used as host plants to assess differential gene expression in *A. gossypii* post-acquisition of CLRDV by transcriptome analyses. This study hypothesized that aphid genes will be differentially expressed when they acquire the same virus isolate from different host species, and consequently aphid behavior and fitness could be differentially affected. The cDNA libraries were prepared for viruliferous and non-viruliferous *A. gossypii* after a 72-h acquisition access period (AAP) on CLRDV-infected or non-infected host plants. The specific objectives of this study were to i) assess the differences in gene expression in *A. gossypii* upon virus acquisition from four CLRDV-infected hosts and ii) locate putative hub genes and co-expressed genes (modules) in *A. gossypii* post-acquisition of CLRDV from specific hosts using weighted gene correlation network analysis (WGCNA).

## Materials and methods

### Plants and insects

The CLRDV host species identified in a previous study, viz., cotton, *Gossypium hirsutum* L. cv. PHY 339 WRF (Corteva, Indianapolis, IN, USA), hibiscus, *H. acetosella* Welw. Ex Hiern. (Johnny’s Selected Seeds, Winslow, ME, USA), okra, *A. esculentus* L. cv. ‘Clemson spineless 80’ (Clemson University, Clemson, SC, USA), and prickly sida, *S. spinosa* L. (Azlin Seed Service, Leland, MS, USA) were used as inoculum sources in this study (Pandey et al., 2022). Two to four seeds of each plant were sown per pot in Sunshine propagation mix (SunGro Horticulture Industries, Bellevue, WA, USA) in 10-cm-diameter plastic pots (depth 8 cm). The pots were kept in insect-proof cages of size 47.5 (l) × 47.5 (w) × 93 (h) cm^3^ (Megaview Science Co., Taichung, Taiwan) in the greenhouse. The greenhouse was maintained at 25°C, 60% relative humidity, and 14-h L:10-h D photoperiod. The seedlings were thinned post-germination, and only one plant per pot was used. Water-soluble Miracle-Gro (Scotts Miracle-Gro Products, Inc., Marysville, OH, USA) at 0.5 g/L was used for weekly fertilization. The aphids were originally collected from cotton fields in 2017 at Tifton, Georgia, and thereafter, the population was maintained in the greenhouse at the University of Georgia, Griffin Campus, under the same conditions indicated above.

### Maintenance of CLRDV-infected plants

Cotton plants infected with CLRDV were originally collected from cotton fields in September 2020 at the University of Georgia, Tifton Campus, GA, USA, and maintained in the greenhouse at the above-stated conditions. Aphid-mediated CLRDV transmission to cotton seedlings was undertaken to maintain the virus inoculum source. CLRDV-infected cotton, hibiscus, okra, and prickly sida plants were obtained following the protocols described in an earlier study (Pandey et al., 2022). *A. gossypii* adults were provided with a 72-h AAP on CLRDV-infected cotton plants followed by a 72-h inoculation access period (IAP) on the undersurface of leaves of young seedlings at the two-true leaf stage. The CLRDV-inoculated plants were placed in aphid-proof cages under the greenhouse conditions described above. Clip cages and aphids were removed 3 days post-inoculation. The infection status of the plants was evaluated at approximately 3 weeks post-inoculation by reverse transcription–PCR as described earlier (Pandey et al., 2022).

### Viruliferous and non-viruliferous aphids for RNA sequencing

**Figure 1:**
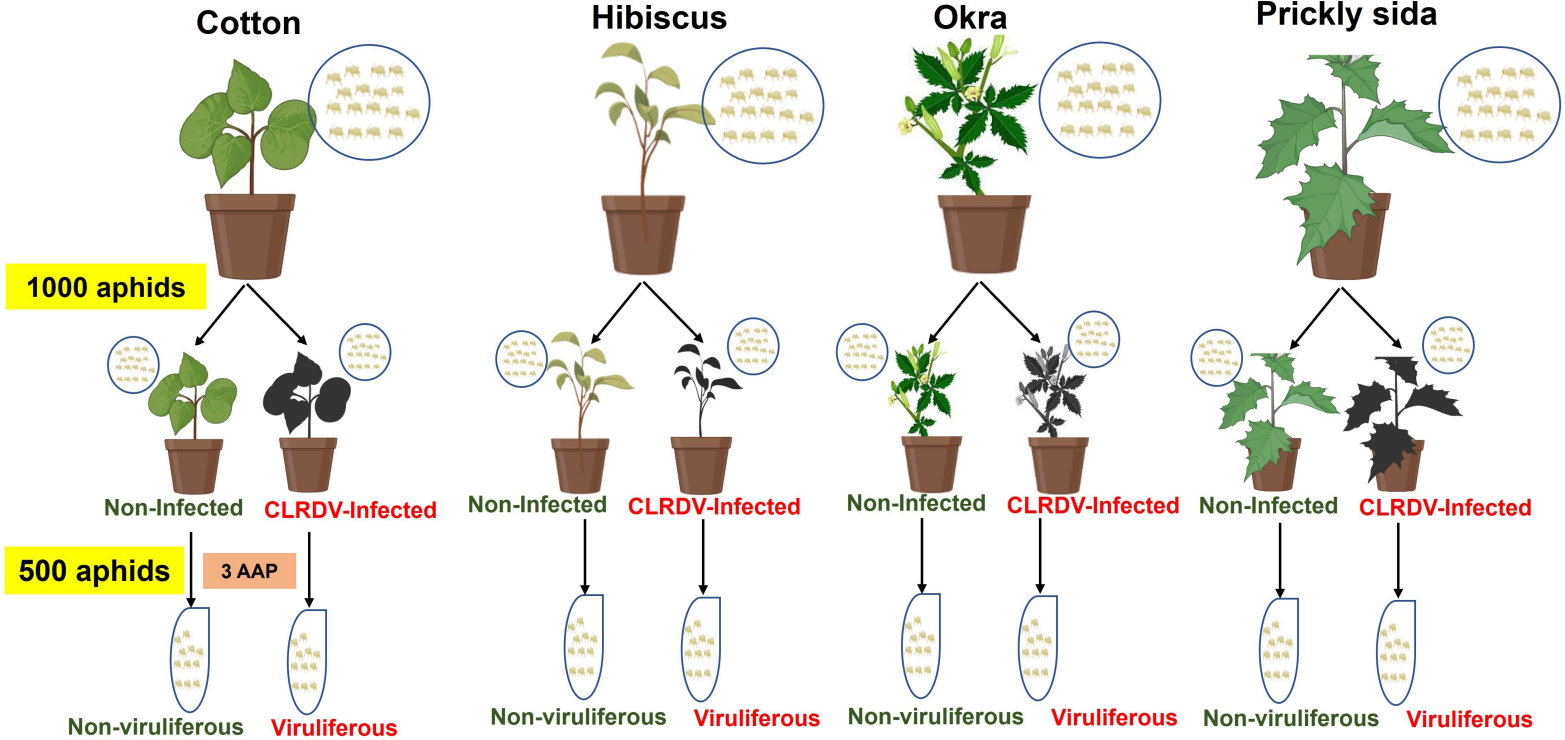
Schematic diagram of experimental setup for generating viruliferous and non-viruliferous aphid samples from CLRDV-infected and non-infected cotton, hibiscus, okra, and prickly sida plants. The aphid colonies were maintained on each plant species separately, and 1,000 adult aphids from each colony were collected and attached to the respective CLRDV-infected and non-infected plants. After 3 days of acquisition access period (AAP), 500 aphids from each treatment were collected for total RNA extraction. The experiment was repeated four times to obtain eight aphid samples from each host plant. CLRDV, cotton leafroll dwarf virus.

The non-viruliferous *A. gossypii* colonies were maintained on all four host species, i.e., non-infected cotton, hibiscus, okra, and prickly sida plants, in separate insect-proof cages. Approximately 1,000 *A. gossypii* were collected from each colony and then introduced to non-infected or CLRDV-infected cotton, hibiscus, okra, and prickly sida plants. The aphids were allowed to feed for an AAP of 72 h. After 72 h of AAP on non-infected or CLRDV-infected plants, approximately 500 adults per treatment were collected, and total RNA was extracted from the collected aphid samples (**Figure 1**). Each treatment was biologically replicated four times. Another set of aphids (three pools of 10 aphids/per treatment) was used to confirm the acquisition of CLRDV in different treatments. The results indicated that 100% of pools of aphids collected from CLRDV-infected plants were positive for CLRDV, whereas aphids feeding on non-infected plants tested negative for CLRDV.

### Total RNA extraction and sequencing

The total RNA was extracted from the collected aphid samples using Qiagen RNA mini-Kit (Valencia, CA, USA) as per the manufacturer’s instructions. For each sample, 40 µL of total RNA was shipped to Novogene Corporation Inc. (Sacramento, CA, USA), and the rest of the total RNA was stored at −80°C for validation. Quality control (QC) of RNA samples was accomplished by preliminary quantitation using a NanoDrop and testing for RNA degradation and contamination via agarose gel electrophoresis. Then, RNA integrity (RIN) was assessed using Agilent 2100. Three samples failed the QC test. The samples that passed QC (RIN value >6.8 and concentration of >20 ng/μL) were used for cDNA library preparation. The library preparation began with enriching mRNA using oligo(dT) beads and removing rRNA using the Ribo-Zero kit. Then, the mRNA fragmentation was followed by first- and second-strand cDNA synthesis. Subsequently, adaptor ligation and PCR enrichment were performed for cDNA library generation. Finally, the library quality was assessed using a Qubit 2.0. NovaSeq 6000 Sequencing System (Illumina, San Diego, CA, USA) with the NovaSeq paired-end 150 sequencing platform was used for sequencing the libraries that passed QC.

### Transcriptome assembly and analysis

FastQC v0.11.9 and multiQC v1.11 were used to assess the quality of raw reads before and after trimming (Andrews and FastQC, 2010; Ewels et al., 2016). The adapters were removed by using Trimmomatic v0.39 with the default setting (Bolger et al., 2014). Bowtie2 v2.4.1 was used with default mapping parameters to map the trimmed reads with the reference *A. gossypii* transcriptome (Langmead and Salzberg, 2012; Quan et al., 2019). RSEM v1.3.3 was used to obtain gene count estimates of the mapped reads (Li and Dewey, 2011). Fragments per kilobase million (FPKM) were determined using a custom R script with the following R libraries: dplyr, tidyverse, and stringr on R v4.1.0 (R Core Team, 2021). DESeq2 compared the gene counts from non-viruliferous aphids with viruliferous aphids to identify DEGs. Genes that had log2fold changes |LFC| ≥ 1 and a false discovery rate (FDR) ≤0.05 were identified as DEGs (Love et al., 2014). The DEGs were annotated and assigned to gene ontology (GO) classes (up to level 3) and Kyoto Encyclopedia of Genes and Genomes (KEGG) pathways (Kanehisa and Goto, 2000) using the annotated *A. gossypii* genome (Quan et al., 2019). TopGO (https://www.bioconductor.org/packages/release/bioc/html/topGO.html) and visualize Gene Ontology (REVIGO) web tool were used for the processing and visualization of the GO terms (Supek et al., 2011).

The WGCNA software v1.70-3 was run to create co-expression modules and identify sets of DEGs expressed in a similar pattern in *A. gossypii* that acquired the CLRDV from alternate hosts using the R software v4.1.0 (Langfelder and Horvath, 2008; R Core Team, 2021). The gradient-independent method with the scale-independent condition of the signed R2 set to 0.90 was used to test the soft-thresholding power modules (1 to 40). The topological overlap matrix (TOM) was constructed using the interaction relationships across the co-expression modules by using correlation expression values. A dendrogram with the parameters mergeCutHeight = 0.15 and detectCutHeight = 0.995 was set to represent the TOM. Each module was represented using randomly assigned colors. The module eigengene was calculated from the first principal component of each module. The labeledHeatmap package in WGCNA software was used to show the topological overlap of co-expression modules based on eigengenes. The network analysis of the top 30 genes from the *A. gossypii* turquoise was visualized using Cytoscape v3.9.0 (Shannon et al., 2003). Also, the hub genes in the most correlated clusters (magenta, pink, brown, and gray) observed in viruliferous *A. gossypii* that acquired the virus from each host species (cotton, hibiscus, okra, and prickly sida) were visualized using Cytoscape v3.9.0.

### Validation of RNA-sequencing data by RT-qPCR

To validate transcriptomic data, 10 DEGs of *A. gossypii* were randomly selected for each host species (n = 40). The expression levels of the DEGs were compared between viruliferous and non-viruliferous *A. gossypii* that acquired CLRDV from different hosts (cotton, hibiscus, okra, and prickly sida) by RT–quantitative polymerase chain reaction (qPCR). Primer pairs designed for each DEG using Primer3web version 4.1.0 are listed in **Supplementary Table S1**. The GoScript™ Reverse Transcription System (Promega, Madison, WI, USA) was used to reverse-transcribe the total RNA for each sample according to the manufacturer’s instructions. Then, the cDNA was diluted 20-fold for qPCR. The 2xGoTaq^®^ qPCR Master Mix (7.5 µL) (Promega, Madison, WI, USA), primers (0.3 µM), 1 µL of cDNA, and nuclease-free distilled water for a final volume of 15 µL were mixed. The QuantStudio™ 3 Real-Time PCR System (Applied Biosystems by Thermo Fisher Scientific, Waltham, MA, USA) was used for qPCR. The following qPCR conditions were used: an initial denaturation step at 95°C for 3 minutes followed by 40 cycles at 95°C for 15 s and 60°C for 1 minute. Three technical replicates for each sample were used, and the melting curve analysis was conducted to evaluate the specificity of the fluorescence signal. The expression level of each gene was normalized to the expression level of elongation factor 1α (EF1α)—an *A. gossypii* reference gene. The 2^−ΔΔCt^ method was used to calculate the relative expression of DEGs (Ma et al., 2016).

## Results

### Summary of RNA sequencing

Three to four biological replicates were included per treatment on each host, resulting in seven cDNA libraries constructed for *A. gossypii* on each host (cotton, hibiscus, and prickly sida plants), whereas eight libraries were constructed for *A. gossypii* on okra. Hence, a total of 29 libraries were constructed. Raw read pairs for the generated libraries ranged from nearly 19 to 34 million. After trimming and removing the reads that aligned with the ribosomal RNA and the mitochondrial genome, 19 to 33 million reads were retained (**Table 1**). *A. gossypii* cleaned read pairs from different libraries (63 to 82%) were mapped to the *A. gossypii* transcriptome (**Table 1**).

**Table 1:** Summary of RNA-sequencing datasets generated from *Aphis gossypii* adults provided with feeding access for 72 h on cotton leafroll dwarf virus-infected or non-infected cotton, hibiscus, okra, and prickly sida plants.

Host plant	Aphid sample description	Library ID	No. of raw read pairs	No. final cleaned read pairs	No. mapped	% Mapped
**Cotton**	Viruliferous rep 1	VCA3	21,945,977	21,713,280	15,207,354	71.32
	Viruliferous rep 2	VCA31	22,410,682	22,186,141	15,823,387	72.96
	Viruliferous rep 3	VCA4	23,484,566	21,083,698	16,966,911	82.88
	Non-viruliferous 1	NCA1	21,411,742	21,173,955	14,922,104	70.47
	Non-viruliferous 2	NCA21	20,444,716	20,241,718	14,728,795	72.76
	Non-viruliferous 3	NCA31	25,859,605	25,586,107	18,430,062	72.03
	Non-viruliferous 4	NCA4	21,551,498	21,321,979	15,479,092	72.60
**Hibiscus**	Viruliferous rep 1	VHA2	21,750,272	21,535,344	14,776,609	68.62
	Viruliferous rep 2	VHA31	21,073,589	20,863,690	15,371,030	73.67
	Viruliferous rep 3	VHA41	22,643,895	22,416,676	16,318,387	72.80
	Non-viruliferous 1	NHA1	19,760,059	19,579,065	14,062,884	71.83
	Non-viruliferous 2	NHA2	22,087,712	21,852,903	15,802,753	72.31
	Non-viruliferous 3	NHA31	22,047,340	21,835,392	15,627,346	71.57
	Non-viruliferous 4	NHA41	29,645,536	29,332,261	20,541,833	70.03
**Okra**	Viruliferous rep 1	VOA5	29,963,400	29,514,383	20,328,303	68.88
	Viruliferous rep 2	VOA6	26,044,703	25,668,773	18,363,422	71.54
	Viruliferous rep 3	VOA7	31,133,707	30,628,153	20,935,316	68.35
	Viruliferous rep 4	VOA8	28,946,216	28,511,952	20,019,531	70.21
	Non-viruliferous 1	NOA11	21,383,934	21,171,951	14,256,767	67.34
	Non-viruliferous 2	NOA2	21,834,007	21,609,657	15,320,142	70.89
	Non-viruliferous 3	NOA31	20,943,829	20,717,474	14,232,750	68.70
	Non-viruliferous 4	NOA4	22,327,129	22,097,040	15,290,654	69.20
**Prickly sida**	Viruliferous rep 1	VTA11	30,962,491	30,453,700	19,301,698	63.38
	Viruliferous rep 2	VTA2	32,573,331	32,067,776	21,748,225	67.82
	Viruliferous rep 3	VTA3	26,690,046	26,330,120	17,217,266	65.39
	Viruliferous rep 4	VTA41	31,546,741	31,118,783	20,631,968	66.30
	Non-viruliferous 1	NTA11	26,883,664	26,525,060	18,276,409	68.90
	Non-viruliferous 2	NTA21	34,473,691	33,950,452	22,458,131	66.15
	Non-viruliferous 3	NTA31	27,134,539	26,748,727	18,013,818	67.34

The reads obtained from viruliferous and non-viruliferous *A. gossypii* samples from different host species were normalized and clustered using FPKM and principal component analysis (PCA) for comparison. The PCA clustered viruliferous *A. gossypii* samples separately from the non-viruliferous samples for all four host species (**Supplementary Figures S1A–D**).

### Overview of DEGs

**Figure 2:**
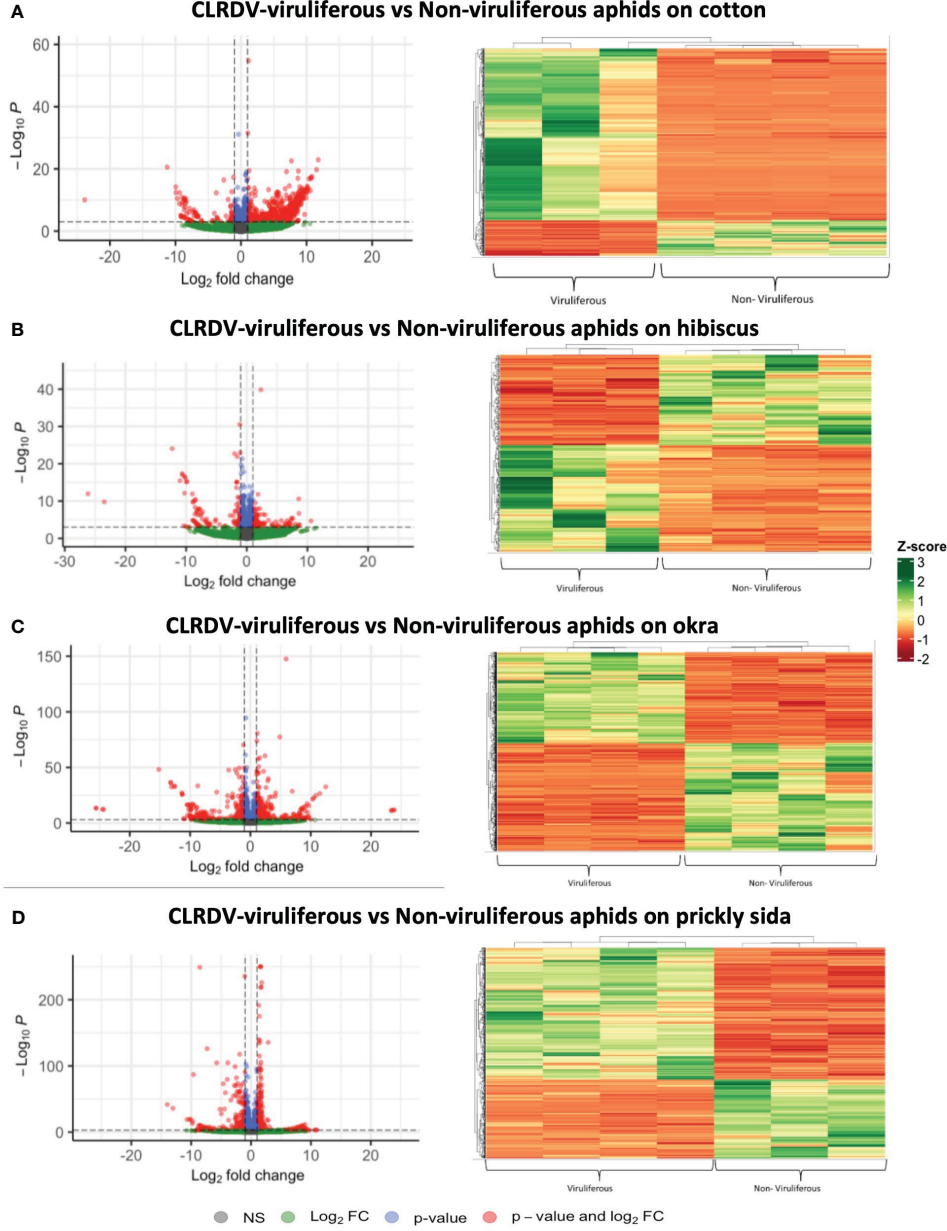
Left: Volcano plots detailing the differential expression profiles of CLRDV-viruliferous versus non-viruliferous *Aphis gossypii*. Genes with an |LFC| ≥ 1 and a false discovery rate (FDR) <0.05 are highlighted in red and were differentially expressed. Right: Hierarchical clustering analysis of normalized count data z-scores exhibited by differentially expressed genes: **(A)** 750 DEGs in viruliferous *A. gossypii* adults that acquired CLRDV from infected cotton plants, **(B)** 310 DEGs in viruliferous *A*. *gossypii* adults that acquired CLRDV from infected hibiscus plants, **(C)** 1,193 DEGs in viruliferous *A*. *gossypii* adults that acquired CLRDV from infected okra plants, and **(D)** 689 DEGs in viruliferous *A*. *gossypii* adults that acquired CLRDV from infected prickly sida plants. CLRDV, cotton leafroll dwarf virus; DEGs, differentially expressed genes.

Out of 14,134 annotated genes, a total of 750 (622 overexpressed and 128 underexpressed), 310 (168 overexpressed and 142 underexpressed), 1,193 (548 overexpressed and 645 underexpressed), and 689 genes (432 overexpressed and 257 underexpressed) were differentially expressed in viruliferous aphids that acquired CLRDV from infected cotton, hibiscus, okra, and prickly sida plants, respectively (**Figure 2**; **Supplementary Figure S2**).

RT-qPCR with 10 randomly selected DEGs per *A. gossypii* host (n = 40) was performed to validate RNA sequencing-based differential gene expression results (**Supplementary Table S1**). The expression trends of the randomly selected *A. gossypii* DEGs from RNA sequencing and RT-qPCR were highly consistent for all four host species (**Supplementary Figures S3A–D**).

### Common DEGs among viruliferous *A. gossypii* adults feeding on different host species

**Figure 3:**
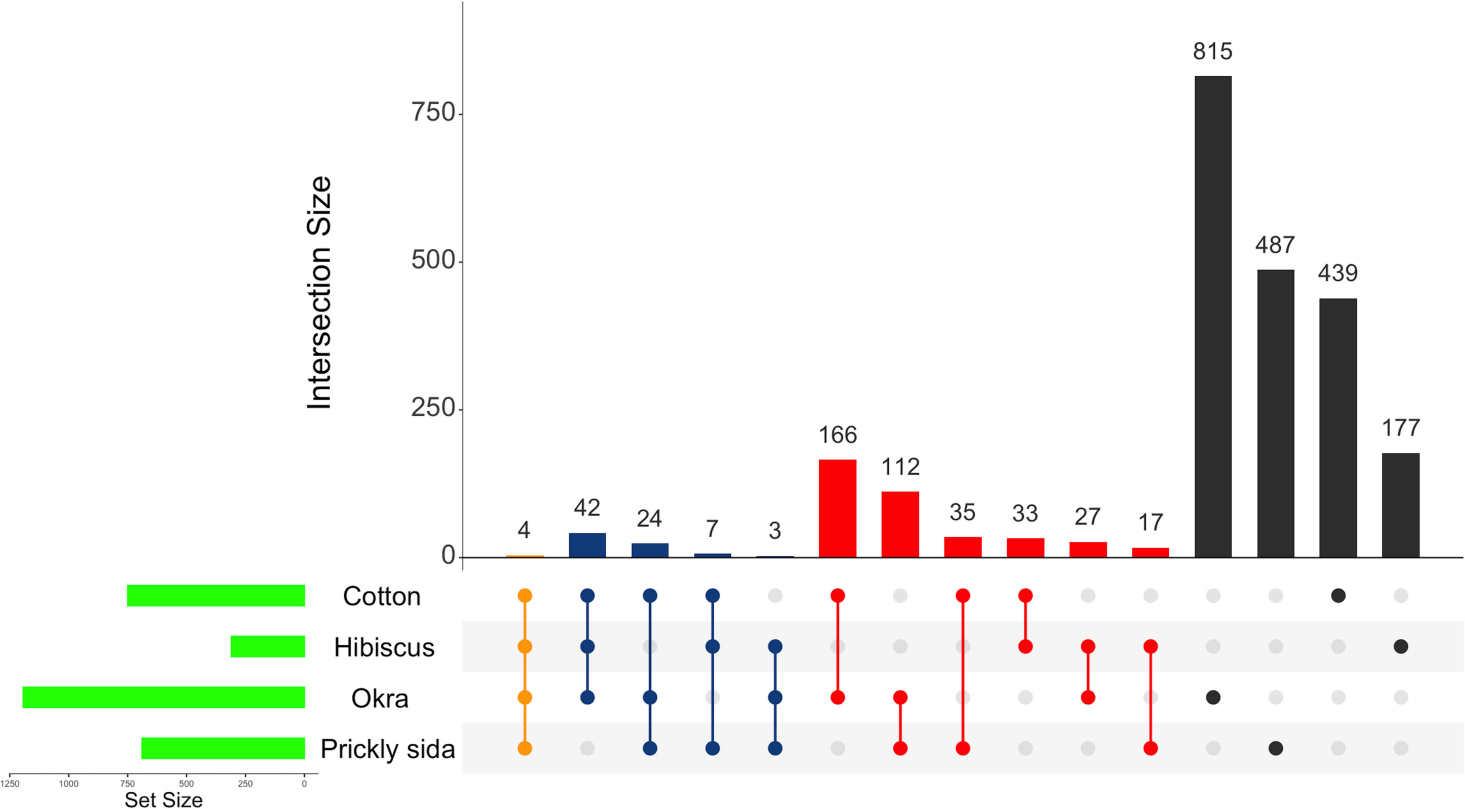
Normalized Venn diagram showing unique and common DEGs in viruliferous *Aphis gossypii* adults that acquired CLRDV from infected cotton, hibiscus, okra, and prickly sida plants. DEGs, differentially expressed genes; CLRDV, cotton leafroll dwarf virus.

Of the 2,942 DEGs in aphids that acquired CLRDV from different host species, only four genes were found to be differentially expressed in common (**Figure 3**). Two common DEGs were annotated as uncategorized proteins, whereas the other two were functionally annotated. Both the unknown DEGs were overexpressed in *A. gossypii* on all four hosts, whereas differences in the direction of expression were observed for the annotated common DEGs. The annotated common DEGs were *juvenile hormone acid O-methyltransferase* and *heat shock protein*. They were overexpressed in *A. gossypii* that acquired the virus from CLRDV-infected cotton and okra, whereas they were underexpressed in *A. gossypii* that acquired CLRDV from infected hibiscus and prickly sida (**Table 2**).

**Table 2:** List of common DEGs in *Aphis gossypii* adults that acquired CLRDV from the infected cotton, hibiscus, okra, and prickly sida plants.

Common DEGs	Annotation	LFC in *A. gossypii* genes acquiring the virus from			
		Cotton	Hibiscus	Okra	Prickly sida
XM_027995716.1	Uncharacterized protein LOC114130688	1.63	1.72	2.51	2.85
XM_027991388.1	Uncharacterized protein LOC114127180	2.15	1.22	1.14	1.78
XM_027987236.1	Juvenile hormone acid *O*-methyltransferase	2.35	−1.79	2.15	−3.25
XM_027981156.1	Heat shock protein 70	2.04	−1.02	4.18	−2.20

The negative sign indicates the underexpressed DEGs, whereas no negative sign indicates the overexpressed DEGs.

DEGs, differentially expressed genes; CLRDV, cotton leafroll dwarf virus.

### Functional annotation of DEGs

**Figure 4:**
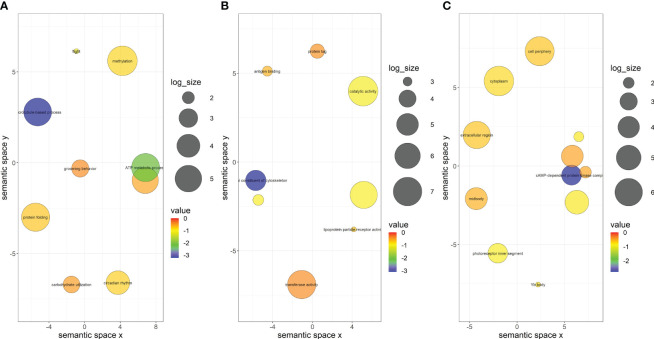
Scatterplots showing **(A)** biological process, **(B)** cellular component, and **(C)** molecular function gene ontology terms in viruliferous *Aphis gossypii* adults that acquired CLRDV from infected cotton plant. Cluster representatives in a two-dimensional space were derived by applying multidimensional scaling to a matrix of the semantic similarities of the gene ontology terms. The bubble color indicates the p-value, and the size indicates the frequency of the GO term in the underlying GOA database. CLRDV, cotton leafroll dwarf virus; GO, gene ontology; GOA, Gene Ontology Annotation.

Only 320 of the 750 DEGs in *A. gossypii* that acquired CLRDV from infected cotton were assigned functional groups under three classification systems: biological process (313 genes), molecular function (267 genes), and cellular component (287 genes). Fifty-three GO terms were assigned under the biological process category, of which only two terms (microtubule-based process and ATP metabolic process) were significant (**Figure 4A**; **Supplementary Table S2**). Thirty-five GO terms were assigned under the molecular function category, only one (a structural constituent of the cytoskeleton) of which was significant (**Figure 4B**; **Supplementary Table S2**). In the cellular component category, 30 GO terms were identified, and only one (cAMP-dependent protein kinase complex) was significant (**Figure 4C**; **Supplementary Table S2**). Similarly, the DEGs identified in aphids that acquired CLRDV from hibiscus, okra, and prickly sida were assigned functional groups under three classification systems (**Supplementary Figures S4**–**S6**; **Supplementary Tables S3**-**S5**). The categorization of these genes was used to identify the DEGs associated with virus–vector interactions.

### Co-expression networks from *A. gossypii* on CLRDV hosts

**Figure 5:**
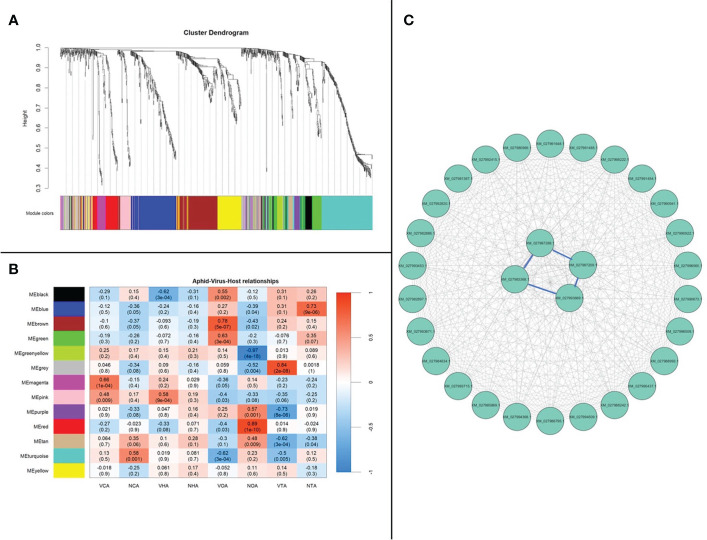
*Aphis gossypii* adults weighted gene co-expression network analysis. **(A)** Dendrogram clustering shows eight modules of co-expressed genes. A total of 981 genes are represented in this network, with 190 genes belonging to MEturquoise. **(B)** Heatmap showing the correlation of module eigengenes in relation to *A*. *gossypii* that acquired CLRDV from cotton, hibiscus, okra, and prickly sida. **(C)** Top 30 genes from MEturquoise with connectivity lines (blue) associated with the top 5% of the connected genes. NCA, non-viruliferous aphid from cotton; VCA, viruliferous aphid from cotton; NHA, non-viruliferous aphid from hibiscus; VHA, viruliferous aphid from hibiscus; NOA, non-viruliferous aphid from okra; VOA, viruliferous aphid from okra; NTA, non-viruliferous aphid from prickly sida; VTA, viruliferous aphid from prickly sida; CLRDV, cotton leafroll dwarf virus.

The co-expression of genes from *A. gossypii* adults that acquired CLRDV from cotton, hibiscus, okra, and prickly sida was evaluated. WGCNA, which clusters genes into modules based on weighted gene–gene interactions, was used to evaluate co-expression. For *A. gossypii*, 13 modules with 19 to 180 genes in each module were identified (**Figure 5A**; **Supplementary Table S6**), and Pearson’s correlation coefficient analysis showed the connections between the four CLRDV hosts. The heatmap visualized overall patterns of co-expression of (viruliferous/non-viruliferous) aphid–host relationships (**Figure 5B**). *A. gossypii* interactions for the largest module, MEturquoise, were checked for top interacting genes among the 30 identified genes (**Figure 5C**). The top four most highly connected genes were XM_027993669.1 (*trichohyalin*-like), XM_027997289.1 (uncharacterized protein), XM_027997209.1 (*glucose dehydrogenase* [*FAD*, *quinone*]-like), and XM_027983368.1 (*neuroendocrine convertase 1*-like) with interconnectivity scores of 58.99, 57.81, 53.87, and 52.58, respectively (**Supplementary Table S6**).

### Hub genes from candidate modules

Four modules (magenta, pink, brown, and gray) were highly correlated with viruliferous *A. gossypii* that acquired CLRDV from infected cotton, hibiscus, okra, and prickly sida plants, respectively. The magenta module associated with viruliferous *A. gossypii* that acquired CLRDV from infected cotton plants contained 34 genes. Similarly, pink, brown, and gray modules associated with *A. gossypii* that acquired CLRDV from infected hibiscus, okra, and prickly sida plants included 40, 130, and 129 genes, respectively. The maximum connectivity in magenta, pink, and brown modules were 8.9, 8.8, and 20, respectively. However, the connectivity in the gray module was less than one. Hence, only magenta, pink, and brown modules were considered to identify candidate genes related to virus interactions and transmission (**Supplementary Table S6**).

**Figure 6:**
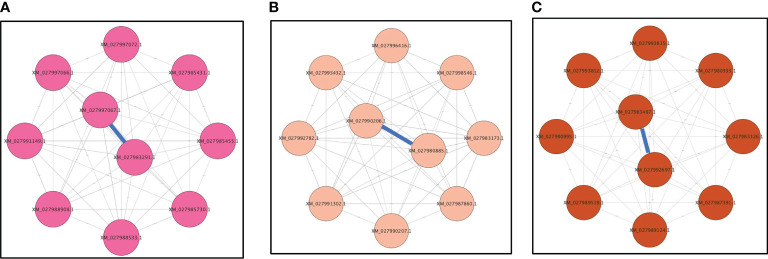
Top 10 genes from **(A)** magenta, **(B)** pink, and **(C)** brown modules with connectivity lines (blue) associated with viruliferous *Aphis gossypii* adults that acquired CLRDV from infected cotton, hibiscus, and okra plants, respectively. CLRDV, cotton leafroll dwarf virus.

Most genes in the magenta module were overexpressed only in viruliferous *A. gossypii* that acquired CLRDV from infected cotton plants, and 10 genes were identified as hub genes based on their high connectivity values (**Figure 6A**). Some of the hub genes were predicted to encode *tubulin-β* (XM_027988533.1 and XM_027985455.1), *tubulin-α* (XM_027988908.1), *GPI-anchored protein* (XM_027997066.1), *U1 small nuclear ribonucleoprotein* (XM_027985431.1), *mucin-7-like* (XM_027997067.1), and *dynein beta chain* (XM_027985730.1) (**Supplementary Table S6**). The *tubulin-α* and *tubulin-β* genes were associated with cellular responses following virus acquisition (**Table 3**).

**Table 3:** Differential expression of genes associated with cellular responses (endocytosis, apoptosis, lysosome, and phagosome) in viruliferous *Aphis gossypii* adults compared with non-viruliferous adults.

Gene ID	Function	LFC of *A. gossypii* genes acquiring the virus from			
		Cotton	Hibiscus	Okra	Prickly sida
XM_027981156.1	Heat shock protein 70	2.04	−1.02	4.18	−2.20
XM_027981452.1	Formylglycine-generating enzyme			1.10	
XM_027981875.1	Proton-coupled amino acid transporter-like protein CG1139			9.64	
XM_027981872.1	Proton-coupled amino acid transporter-like protein CG1139			−9.21	
XM_027982644.1	Dynamin-1-like protein	6.88			
XM_027985455.1	Tubulin beta chain	5.48		−10.53	
XM_027985527.1	GSK3B-interacting protein-			−6.51	
XM_027985860.1	Tubulin beta-1 chain	7.71			
XM_027986526.1	Proton-coupled amino acid transporter-like protein CG1139			−1.37	
XM_027987245.1	Sialin-like	8.62		−6.68	
XM_027988908.1	Tubulin alpha-4 chain	8.22		−11.06	
XM_027990131.1	Alpha-l-fucosidase		7.38		
XM_027992363.1	ADP-ribosylation factor-binding protein GGA1			−9.79	
XM_027992365.1	ADP-ribosylation factor-binding protein GGA1			1.34	
XM_027992845.1	Phosphatidylinositol 3,4,5-trisphosphate 3-phosphatase and dual-specificity protein phosphatase PTEN			1.17	
XM_027993821.1	Mitochondrial Rho GTPase			1.02	
XM_027994292.1	Lipopolysaccharide-induced tumor necrosis factor-alpha factor homolog				1.41
XM_027994521.1	Phosphatidylinositol 3-kinase regulatory subunit gamma			−8.20	
XM_027995767.1	Proton-coupled amino acid transporter-like protein CG1139			1.03	
XM_027996300.1	Protein transport protein Sec61 subunit gamma			−1.14	
XM_027996848.1	Actin-42A	1.68			−2.55
XM_027997003.1	Heat shock protein 68			4.87	−2.38
XM_027997432.1	Heat shock protein 70	2.04		4.13	
XM_027998191.1	Oxidation resistance protein 1				1.17
XM_027998701.1	Heat shock protein 70	1.90		3.48	−1.02

Genes with the same annotation name but different gene IDs are isoforms. The negative sign indicates the underexpressed DEGs, whereas no negative sign indicates the overexpressed DEGs.

DEGs, differentially expressed genes.

Similarly, most genes in the pink module were underexpressed in viruliferous *A. gossypii* that acquired CLRDV from infected hibiscus plants, and 10 genes were identified as hub genes based on their high connectivity values (**Figure 6B**). Some of these hub genes were predicted to encode *proteasome subunit-β* (XM_027980885.1), *trehalose transporter Tret1* (XM_027996416.1), and *M-phase inducer phosphatase* (XM_027998546.1) (**Supplementary Table S6**).

Most genes in the brown module were underexpressed in viruliferous *A. gossypii* that acquired CLRDV from infected okra plants, and 10 genes were identified as hub genes based on their high connectivity values (**Figure 6C**). These hub genes were predicted to encode ubiquitin *carboxyl-terminal hydrolase 7* (XM_027983467.1), *dynein heavy chain* (XM_027987391.1), *serine/threonine-protein kinase WNK1* (XM_027980933.1), and *eukaryotic translation initiation factor 4 gamma* (XM_027992697.1) (**Supplementary Table S6**). The *serine/threonine-protein kinase* genes were associated with signal transduction following virus acquisition (**Table 4**).

**Table 4:** Differential expression of genes associated with signal transduction in viruliferous *Aphis gossypii* adults compared with non-viruliferous adults.

Gene ID	Function	LFC of *A. gossypii* genes acquiring the virus from			
		Cotton	Hibiscus	Okra	Prickly sida
XM_027980601.1	Acyl-CoA Delta(11) desaturase			1.35	
XM_027981156.1	Heat shock protein 70	2.04	−1.02	4.18	−2.20
XM_027982467.1	Phosphatidylinositol 4-kinase type 2-beta			1.05	
XM_027982644.1	Dynamin-1-like protein	6.88			
XM_027983386.1	Sodium/potassium-transporting ATPase subunit alpha	−6.52			4.22
XM_027983455.1	ADP, ATP carrier protein 2				−2.55
XM_027983505.1	Probable phosphorylase b kinase regulatory subunit alpha		−8.08		
XM_027983941.1	Serine palmitoyltransferase 2				−1.16
XM_027985527.1	GSK3B-interacting protein			−6.51	
XM_027987983.1	Protein giant-lens-like			1.29	
XM_027988560.1	Guanine nucleotide-binding protein G(o) subunit alpha	−1.33		−1.99	
XM_027989043.1	Plasma membrane calcium-transporting ATPase 2			−1.04	
XM_027989047.1	Plasma membrane calcium-transporting ATPase 2			−1.19	
XM_027989048.1	Plasma membrane calcium-transporting ATPase 2				1.75
XM_027989866.1	Low-molecular-weight phosphotyrosine protein phosphatase		−6.99	−7.91	
XM_027990405.1	Embryonic polarity protein dorsal-like			1.47	
XM_027990451.1	cAMP-dependent protein kinase catalytic subunit beta	7.28			
XM_027990995.1	(11*Z*)-Hexadec-11-enoyl-CoA conjugase			−1.11	
XM_027991215.1	ATP-dependent 6-phosphofructokinase				−1.14
XM_027991280.1	Serine/threonine-protein kinase PLK1				8.40
XM_027991279.1	Serine/threonine-protein kinase PLK1				−7.89
XM_027991716.1	Multidrug resistance-associated protein 1			−1.37	
XM_027992190.1	Putative phosphatidate phosphatase			1.44	
XM_027992685.1	5′-AMP-activated protein kinase subunit gamma			1.26	
XM_027992684.1	5′-AMP-activated protein kinase subunit gamma			−4.51	
XM_027992845.1	Phosphatidylinositol 3,4,5-trisphosphate 3-phosphatase and dual-specificity protein phosphatase PTEN			1.17	
XM_027993702.1	Ras-related protein Rab-2A			3.08	
XM_027994156.1	cAMP-dependent protein kinase catalytic subunit beta	7.05			
XM_027994521.1	Phosphatidylinositol 3-kinase regulatory subunit gamma			−8.20	
XM_027994620.1	Tyrosine-protein kinase Btk29A				−1.15
XM_027994967.1	Profilin		−2.75		
XM_027995400.1	Glyceraldehyde-3-phosphate dehydrogenase	7.49	8.33	−8.16	
XM_027995849.1	Calcineurin subunit B type 2			−9.99	
XM_027995848.1	Calcineurin subunit B type 2			−1.16	
XM_027996465.1	Hexokinase type 2		1.32		
XM_027996848.1	Actin-42A	1.68			−2.55
XM_027996979.1	Interleukin-1 receptor-associated kinase 4-like	−4.39			
XM_027997003.1	Heat shock protein 68			4.87	−2.38
XM_027997371.1	Inositol-trisphosphate 3-kinase homolog				2.00
XM_027997432.1	Heat shock protein 70	2.04		4.13	
XM_027997466.1	Misshapen-like kinase 1		−1.29		
XM_027998688.1	ATP-dependent 6-phosphofructokinase	7.79	6.69		
XM_027998701.1	Heat shock protein 70	1.90		3.48	−1.02

Genes with the same annotation name but different gene IDs are isoforms. The negative sign indicates the underexpressed DEGs, whereas no negative sign indicates the overexpressed DEGs.

DEGs, differentially expressed genes.

### DEGs among *A. gossypii* adults associated with virus–vector interactions

#### Virus infection

In *A. gossypii* adults, DEGs associated with different viruses, including measles virus, coronavirus, human cytomegalovirus, human immunodeficiency virus 1, herpes simplex virus 1, human T-cell leukemia virus 1, human papillomavirus, hepatitis B and C, virus, influenza A virus, and Epstein–Barr virus were identified upon CLRDV acquisition from different host species. Eleven, four, 12, and seven genes associated with virus infection in *A. gossypii* that acquired CLRDV from infected cotton, hibiscus, okra, and prickly sida plants, respectively, were identified. The expression of these DEGs ranged from −26.18- to 7.60-fold (**Table 5**).

**Table 5:** Differential expression of genes associated with virus infection in viruliferous *Aphis gossypii* adults compared with non-viruliferous adults.

Gene ID	Function	LFC in *A. gossypii* genes acquiring the virus from			
		Cotton	Hibiscus	Okra	Prickly sida
XM_027981070.1	60S ribosomal protein L10		−26.18		
XM_027981156.1	Heat shock protein 70 A1-like	2.04	−1.02	4.18	−2.20
XM_027982887.1	Angiotensin-converting enzyme			−1.02	
XM_027983455.1	ADP, ATP carrier protein 2				−2.55
XM_027988560.1	Guanine nucleotide-binding protein G(o) subunit alpha	−1.33		−1.99	
XM_027990206.1	60S ribosomal protein L31	−11.27	−12.31	−15.19	
XM_027990451.1	cAMP-dependent protein kinase catalytic subunit beta	7.28			
XM_027990946.1	Uncharacterized protein LOC114126902				−1.02
XM_027991760.1	Ubiquitin-protein ligase E3A			1.59	
XM_027992845.1	Phosphatidylinositol 3,4,5-trisphosphate 3-phosphatase and dual-specificity protein phosphatase PTEN			1.17	
XM_027994156.1	cAMP-dependent protein kinase catalytic subunit beta	7.05			
XM_027994521.1	Phosphatidylinositol 3-kinase regulatory subunit gamma			−8.20	
XM_027994543.1	Zinc finger protein 436	7.60			
XM_027995723.1	40S ribosomal protein SA	−5.99			
XM_027995849.1	Calcineurin subunit B type 2			−9.99	
XM_027995848.1	Calcineurin subunit B type 2			−1.16	
XM_027996455.1	Serine/arginine-rich splicing factor 1A				−1.27
XM_027996848.1	Actin-42A	1.68			−2.55
XM_027996937.1	Oxysterol-binding protein-related protein 6		−4.49		
XM_027996979.1	Interleukin-1 receptor-associated kinase	−4.39			
XM_027997003.1	Heat shock protein 68			4.87	−2.38
XM_027997432.1	Heat shock protein 70	2.04		4.13	
XM_027998701.1	Heat shock protein 70	1.90		3.48	−1.02

Genes with the same annotation name but different gene IDs are isoforms. The negative sign indicates the underexpressed DEGs, whereas no negative sign indicates the overexpressed DEGs.

DEGs, differentially expressed genes.

#### Signal transduction

**Figure 7:**
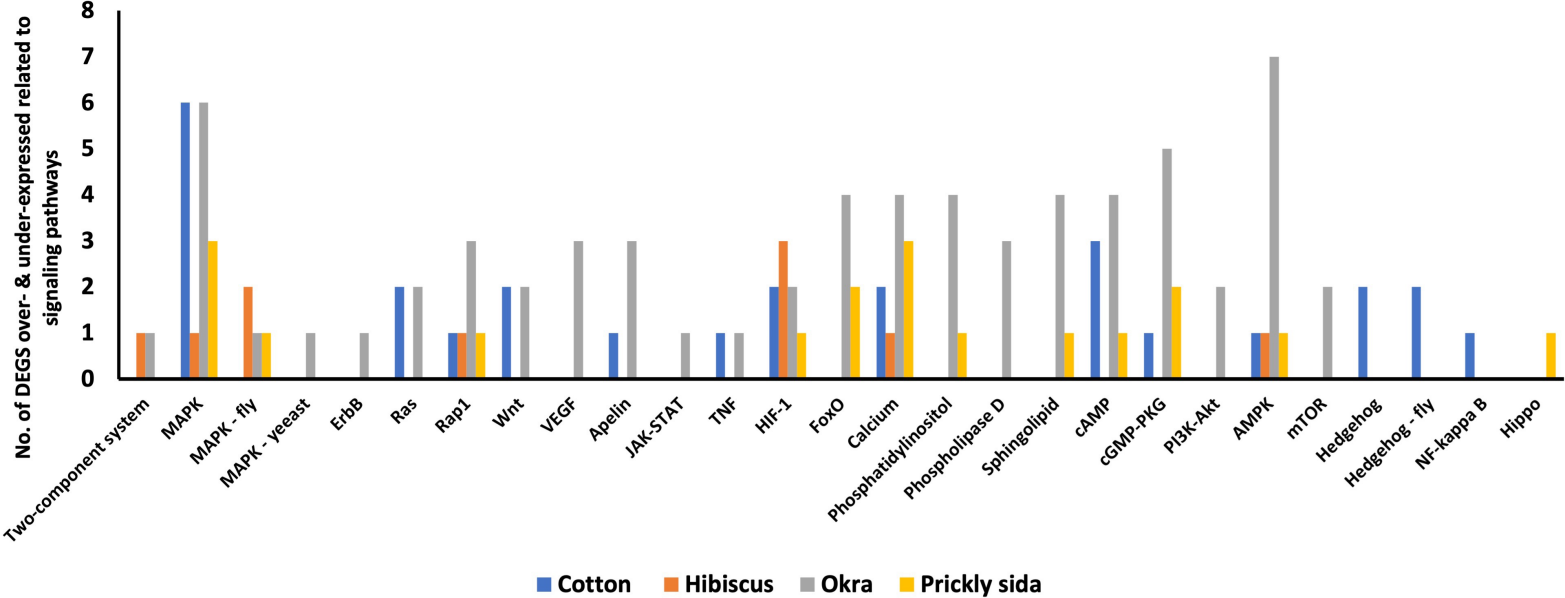
Number of differentially expressed genes (DEGs) related to different signaling pathways in viruliferous *Aphis gossypii* adults that acquired CLRDV from infected cotton, hibiscus, okra, and prickly sida plants. CLRDV, cotton leafroll dwarf virus.

Twelve DEGs were associated with 14 signal transduction pathways in *A. gossypii* that acquired CLRDV from infected cotton plants. Similarly, eight, 24, and 13 DEGs associated with different signal transduction pathways in *A. gossypii* that acquired CLRDV from infected hibiscus, okra, and prickly sida plants, respectively, were identified (**Figure 7**). The expression of these DEGs ranged from −8.08- to 8.40-fold (**Table 4**).

#### Signaling molecules and virus interaction

Using KEGG annotation, one putative receptor gene was identified in *A. gossypii* that acquired CLRDV from infected cotton (XM_027997734.1, *cardioacceleratory peptide receptor*) and one from prickly sida (XM_027980613.1, *neuropeptide SIFamide receptor*). Putative receptor genes were not identified in *A. gossypii* adults that acquired CLRDV from infected hibiscus and okra plants (**Table 6**).

**Table 6:** Differential expression of genes associated with immune systems in viruliferous *Aphis gossypii* adults compared with non-viruliferous adults.

Gene ID	Function	LFC of *A. gossypii* genes acquiring the virus from			
		Cotton	Hibiscus	Okra	Prickly sida
XM_027981156.1	Heat shock protein 70	2.04	−1.02	4.18	−2.20
XM_027982644.1	Dynamin-1-like protein	6.88			
XM_027983455.1	ADP, ATP carrier protein 2				−2.55
XM_027985527.1	GSK3B-interacting protein			−6.51	
XM_027988560.1	Nucleotide-binding protein G(o) subunit alpha	−1.33		−1.99	
XM_027990405.1	Embryonic polarity protein dorsal			1.47	
XM_027990451.1	cAMP-dependent protein kinase catalytic subunit beta	7.28			
XM_027992190.1	Putative phosphatidate phosphatase			1.44	
XM_027994156.1	cAMP-dependent protein kinase catalytic subunit beta	7.05			
XM_027994521.1	Phosphatidylinositol 3-kinase regulatory subunit gamma			−8.20	
XM_027994620.1	Tyrosine-protein kinase Btk29A				−1.15
XM_027994723.1	Histone H3.3		1.41		
XM_027995849.1	Calcineurin subunit B type 2			−9.99	
XM_027995848.1	Calcineurin subunit B type 2			−1.16	
XM_027996455.1	Serine/arginine-rich splicing factor 1A				−1.27
XM_027996848.1	Actin-42A	1.68			−2.55
XM_027996979.1	Interleukin-1 receptor-associated kinase 4-like	−4.39			
XM_027997003.1	Heat shock protein 68			4.87	−2.38
XM_027997432.1	Heat shock protein 70	2.04		4.13	
XM_027998701.1	Heat shock protein 70	1.90		3.48	−1.02

Genes with the same annotation name but different gene IDs are isoforms. The negative sign indicates the underexpressed DEGs, whereas no negative sign indicates the overexpressed DEGs.

DEGs, differentially expressed genes.

#### Immune systems

A total of nine DEGs were annotated using KEGG analysis and associated with six immune system pathways in *A. gossypii* adults that acquired CLRDV from infected cotton plants. Similarly, 11 DEGs with 15 immune system pathways were identified for *A. gossypii* adults that acquired CLRDV from infected okra plants. Only two DEGs from two immune system pathways and seven DEGs from six immune system pathways were identified in *A. gossypii* that acquired CLRDV from infected hibiscus and prickly sida plants, respectively. The expression of these DEGs ranged from 9.99- to 7.28-fold (**Table 6**; **Supplementary Table S7**).

#### Cellular processes (apoptosis, lysosome, and phagosome)

A total of six, one, 15, and three DEGs were associated with cellular processes in *A. gossypii* that acquired CLRDV from infected cotton, hibiscus, okra, and prickly sida plants, respectively. The expression of these DEGs ranged from −11.06- to 9.64-fold (**Table 3**).

In the cellular process category, *heat shock proteins 70* and *68* were identified as genes playing a role in endocytosis. The heat shock proteins were overexpressed in *A. gossypii* acquired CLRDV from infected cotton and okra plants but underexpressed when acquired from infected hibiscus and prickly sida plants. Another gene related to endocytosis was underexpressed in *A. gossypii* that acquired CLRDV from infected okra plants (**Table 7**).

**Table 7:** Differential expression of genes associated with cellular responses (endocytosis) in viruliferous *Aphis gossypii* adults compared with non-viruliferous adults.

Gene ID	Function	LFC of *A. gossypii* genes acquiring the virus from			
		Cotton	Hibiscus	Okra	Prickly sida
XM_027981156.1	Heat shock protein 70	2.04	−1.02	4.18	−2.20
XM_027997432.1	Heat shock protein 70 A1-like	2.04		4.13	
XM_027998701.1	Heat shock protein 70 A1-like	1.89		3.48	−1.0
XM_027985527.1	GSK3B-interacting protein-like			−6.51	
XM_027997003.1	Heat shock protein 68-like			4.87	−2.38

Genes with the same annotation name but different gene IDs are isoforms. The negative sign indicates the underexpressed DEGs, whereas no negative sign indicates the overexpressed DEGs.

DEGs, differentially expressed genes.

### DEGs among *A. gossypii* adults associated with aphid fitness

#### Longevity

Based on KEGG pathway annotation, five DEGs were associated with two aging pathways in *A. gossypii* that acquired CLRDV from infected cotton plants (**Table 8**). Similarly, one, 11, and three DEGs associated with different aging pathways in *A. gossypii* that acquired CLRDV from infected hibiscus, okra, and prickly sida plants, respectively, were identified (**Figure 7**). The expression of these DEGs ranged from −8.49- to 7.28-fold (**Table 8**).

**Table 8:** Differential expression of genes associated with aging in viruliferous *Aphis gossypii* adults compared with non-viruliferous adults.

Gene ID	Function	LFC of *A. gossypii* genes acquiring the virus from			
		Cotton	Hibiscus	Okra	Prickly sida
XM_027980601.1	Acyl-CoA Delta(11) desaturase-like			1.35	
XM_027981156.1	Heat shock protein 70 A1-like	2.04	−1.02	4.18	−2.20
XM_027990451.1	cAMP-dependent protein kinase catalytic subunit beta-like	7.28			
XM_027990995.1	(11*Z*)-Hexadec-11-enoyl-CoA conjugase-like			−1.11	
XM_027990998.1	(11*Z*)-Hexadec-11-enoyl-CoA conjugase-like			−8.49	
XM_027992685.1	5′-AMP-activated protein kinase subunit gamma isoform X1			1.26	
XM_027992684.1	5′-AMP-activated protein kinase subunit gamma isoform X1			−4.51	
XM_027992845.1	Phosphatidylinositol 3,4,5-trisphosphate 3-phosphatase and dual-specificity protein phosphatase PTEN isoform X3			1.17	
XM_027994156.1	cAMP-dependent protein kinase catalytic subunit beta-like	7.05			
XM_027994521.1	Phosphatidylinositol 3-kinase regulatory subunit gamma-like			−8.20	
XM_027997003.1	Heat shock protein 68-like			4.87	−2.38
XM_027997432.1	Heat shock protein 70 A1-like	2.04		4.13	
XM_027998701.1	Heat shock protein 70 A1-like	1.90		3.48	−1.02

Genes with the same annotation name but different gene IDs are isoforms. The negative sign indicates the underexpressed DEGs, whereas no negative sign indicates the overexpressed DEGs.

DEGs, differentially expressed genes.

#### Reproduction

**Figure 8:**
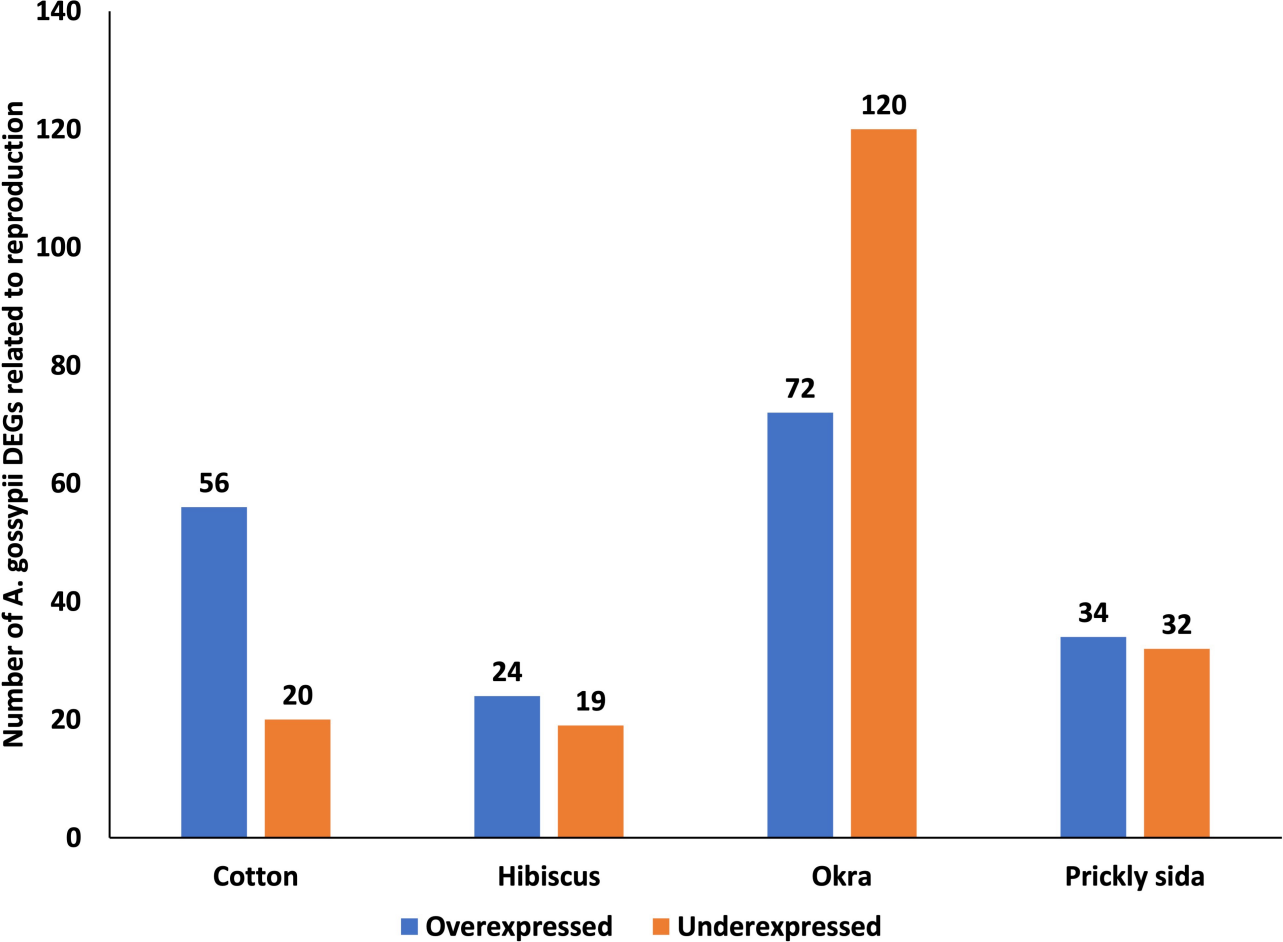
Number of differentially expressed genes (DEGs) related to reproduction in viruliferous *Aphis gossypii* adults that acquired CLRDV from infected cotton, hibiscus, okra, and prickly sida plants. CLRDV, cotton leafroll dwarf virus.

Using GO annotation, several genes associated with reproduction were identified in *A. gossypii* adults. The number of DEGs was the highest in *A. gossypii* that acquired CLRDV from infected okra, followed by cotton, prickly sida, and hibiscus plants (**Figure 8**). Unlike that in *A. gossypii* that acquired CLRDV from infected cotton, hibiscus, and prickly sida plants, the number of underexpressed genes in *A. gossypii* that acquired CLRDV from infected okra plants was higher than the overexpressed genes (**Figure 8**). One of the common genes identified in this GO annotation was *juvenile hormone acid O-methyltransferase* (**Supplementary Table S8**).

## Discussion

In some rare instances, direct effects of persistent non-propagative viruses on their vectors’ behavior and/or fitness have been documented (Bosque-Pérez and Eigenbrode, 2011; Ingwell et al., 2012; Marmonier et al., 2022). However, most effects of persistent and non-propagative phytoviruses on their vectors’ behavior and fitness seem to be modulated by the host plants due to their altered phenotypic traits following virus infection (Eigenbrode et al., 2002; Srinivasan et al., 2006; Ngumbi et al., 2007; Hodge and Powell, 2008; Medina-Ortega et al., 2009; Werner et al., 2009; Bosque-Pérez and Eigenbrode, 2011; Legarrea et al., 2020; Fingu-Mabola and Francis, 2021; Safari Murhububa et al., 2021; Hu et al., 2022). The degree of the altered host phenotype would substantially depend upon the virus susceptibility status of the host. While this phenomenon has been researched in many persistent virus pathosystems involving hemipteran vectors, it has been extremely difficult to parse apart the host effect from the direct virus-induced impacts on vectors (Eigenbrode et al., 2002; Ingwell et al., 2012; Marmonier et al., 2022). In addition, generalizations seem to originate from host and virus-modulated effects on vectors based on individual hosts and viruses that at least possess a modest or often a promiscuous host range (Jiménez-Martínez et al., 2004; Lightle and Lee, 2014; dos Santos et al., 2016; Ghosh et al., 2016; Claudel et al., 2018; Chesnais et al., 2020; Moeini et al., 2020; Bertasello et al., 2021; Fingu-Mabola and Francis, 2021; Jayasinghe et al., 2022). This also applies to vectors and their host utilization capacities when they are generalists (Castle et al., 1998; Eigenbrode et al., 2002; Ngumbi et al., 2007; Rajabaskar et al., 2013; Ren et al., 2015; Liu et al., 2019). Host-modulated virus-induced effects on the vector are realized in the form of ecological, behavioral, and fitness patterns. However, advancements in omics techniques that capture associated gene expression patterns provide greater opportunities to explore this paradigm of vector–virus interactions. This study assessed the differences in gene expression in *A. gossypii* adults in response to the acquisition of CLRDV from its primary host plant (cotton) and alternate host plants (hibiscus, okra, and prickly sida). The results show that transcriptional changes observed in viruliferous *A. gossypii* vary substantially between the host species from which it acquired the virus. The results indicate that the host plant could be a major determinant of vector–virus interaction outcomes.

Across all four host species, most transcriptional changes were observed in *A. gossypii* that acquired CLRDV from infected okra plants, followed by cotton, prickly sida, and hibiscus, which represented 8.11%, 5.1%, 4.6%, and 2.1% of the overall genes in the aphid genome, respectively. Similarly, the number of unique genes of *A. gossypii* was the highest when the virus was acquired from okra, followed by prickly sida, cotton, and hibiscus plants. These findings indicate that CLRDV-induced transcriptional changes in *A. gossypii* adults upon virus acquisition from different host species vary drastically. This variation in the number of transcriptional responses occurring in *A. gossypii* could be affected by differences in host susceptibility to the virus, host nutrient quality, physiology, and defense mechanisms (Gadhave et al., 2019; Marmonier et al., 2022). In a previous study, the percentage of adult aphids that acquired CLRDV, the amount of virus acquired, and the percentage of aphid-mediated back-transmission of the virus varied significantly between the four host plants (Pandey et al., 2022). The transcriptional differences observed when adult *A. gossypii* acquired CLRDV from different hosts observed in this study, in part, could explain some of the observed variations in virus acquisition and inoculation ability of adult aphids (Pandey et al., 2022).

Previous studies also have reported varying transcriptional responses in aphids upon polerovirus acquisition. For instance, 164 DEGs were identified in *M. persicae* adults that acquired TuYV from infected plants compared with non-viruliferous aphids, whereas 201 DEGs were identified when the aphids acquired the virus from an artificial medium compared with non-viruliferous aphids in the same study (Marmonier et al., 2022). Similarly, the number of DEGs was greater in viruliferous aphids that acquired TuYV from *Arabidopsis thaliana* (L.) Heynh (1,073 genes) compared with viruliferous aphids that acquired TuYV from *Camelina sativa* (L.) Crantz (474 genes) (Chesnais et al., 2022). Thus, the variation in the number of DEGs may be influenced by the host species from which it is acquiring the virus. The number of DEGs in the same aphid species (*M. persicae*) upon acquisition of another polerovirus species PLRV from infected potato plants, when compared with their non-viruliferous counterparts, was 134 (Patton et al., 2021). The acquisition of the same virus species (BYDV) from virus-infected wheat plants resulted in significant variation in the number of DEGs of two of its aphid vectors—*S. graminum* (1,525 genes) and *S. avenae* (800 genes)—in comparison with non-viruliferous vectors (Li et al., 2019, 2020). These results reiterate that different viruses and host interactions as well as the same virus–host interactions could differentially induce gene expression in the same vector or different vectors. Also, the experimental design factors such as acquisition period, gut clearing, sequencing platforms, number of libraries sequenced, and bioinformatics tools used for analysis may have contributed to the variation in the number of DEGs across virus–vector–host pathosystems in different studies. What is missing in understanding component interactions within a phytovirus–vector pathosystem is the impact of alternate hosts on virus–vector interactions. In other words, how conserved are vector–virus interactions across host species?

### Common DEGs among viruliferous *A. gossypii* adults associated with virus–vector interactions

In this study, the acquisition of CLRDV resulted in transcriptional changes in *A. gossypii*, of which only four DEGs were common between the viruliferous *A. gossypii* adults acquiring the virus from four host species. Among four common DEGs, the direction and/or level of the expression (over or under) of common genes varied between host species. The KEGG annotation and GO enrichment analysis revealed the role of one of the common genes (XM_027981156.1, *heat shock protein 70*) in virus infection, signal transduction, immune responses, longevity, and endocytosis. Heat shock protein 70 was overexpressed in *A. gossypii* upon acquiring the virus from CLRDV-infected cotton and okra plants, whereas it was underexpressed in *A. gossypii* that acquired the virus from CLRDV-infected hibiscus and prickly sida plants. In this study, the expression level of heat shock protein was nearly double in *A. gossypii* that acquired the virus from infected okra compared with cotton. The heat shock proteins are essential chaperone proteins known to be overexpressed in response to stress conditions. One study found that *heat shock protein 70* was overexpressed upon BYDV acquisition in its aphid vector (*R. padi*). The BYDV infection has been reported to increase the plant surface temperature and aphid heat tolerance, suggesting a protective role (Porras et al., 2020). Another study has reported the interaction of tomato yellow leaf curl virus (TYLCV) with *Bemisia tabaci* (Gennadius) *heat shock protein 70* in the midgut using *in vitro* studies. The protein was suggested to play an inhibitory role in virus transmission (Götz et al., 2012). The higher expression level of heat shock protein could be one of the reasons for reduced CLRDV acquisition and inoculation from okra to cotton plants by *A. gossypii* reported in an earlier study (Pandey et al., 2022).

However, the other common gene (XM_027987236.1, *juvenile hormone acid O-methyltransferase*) was overexpressed in aphids that acquired the virus from cotton and okra but underexpressed in aphids that acquired the virus from hibiscus and prickly sida plants. In a previous study, the *JHAMT* (*juvenile hormone-III synthase*) was overexpressed in *S. avenae* that acquired BYDV from wheat plants (Li et al., 2019). This gene is known to play a regulatory role, as a rate-limiting enzyme in insect juvenile hormone biosynthesis, which is essential in the development, metamorphosis, and reproduction of insects (Shinoda and Itoyama, 2003; Minakuchi et al., 2008; Niwa et al., 2008). In contrast, in another study, the underexpression of juvenile hormones in aphids was linked with increased wing development and enhanced virus spread (Quan et al., 2019; Zhang et al., 2019). Variations in the expression levels of *juvenile hormone acid O-methyltransferase* observed in *A. gossypii* suggest that CLRDV acquisition may enhance or reduce the fitness of *A. gossypii* depending on the host species and warrants further examination.

### Unique DEGs associated with virus–vector interactions

In addition to the four DEGs in common, many unique DEGs were identified in *A. gossypii* depending on the plant species from which the virus was acquired. The number of DEGs uniquely expressed was the highest in aphids that acquired the virus from okra plants. For example, the *ras-related protein* (*Rab* protein) associated with signaling in the circadian clock cells in *Drosophila melanogaster* Meigen was uniquely identified and underexpressed in aphids that acquired the virus from okra plants (Williams et al., 2001). *Rab* proteins also function as transporters of vesicle cargo, responsible for trafficking among several membrane compartments (Zhang et al., 2007). Hence, the underexpression of this gene could be one of the reasons for the lower virus acquisition and/or retention ability of aphids from okra plants. The *tubulin beta-1 chain* gene encoding a structural constituent of the cytoskeleton was uniquely overexpressed in *A. gossypii* that acquired CLRDV from infected cotton plants in this study. The overexpression of this gene enhances the insects’ development and reproduction (Nielsen et al., 2010). Tubulin is also a major constituent of microtubules, which is an integral part of intracellular transport (Logan and Menko, 2019). This could be one of the reasons for better fitness and acquisition of CLRDV in adult *A. gossypii* that acquired the virus from cotton plants compared with the other three hosts in the previous study (Pandey et al., 2022). In contrast, this gene was underexpressed in *M. persicae* adults that acquired TuYV from virus-infected plants and artificial medium (Marmonier et al., 2022).

The gene coding for *ubiquitin-conjugating enzyme* was overexpressed sixfold in *A. gossypii* that acquired CLRDV from virus-infected cotton plants, whereas it was overexpressed ~1.5-fold when the virus was acquired from okra and prickly sida plants in this study. The gene was not differentially expressed in *A. gossypii* acquiring CLRDV from virus-infected hibiscus plants (**Table S3**). The overexpression of *ubiquitin-conjugating enzymes* was previously reported in *M. persicae* and *B. tabaci* feeding on BYDV-infected and sida golden mosaic virus (SiGMV)-infected plants, respectively (Li et al., 2020; Mugerwa et al., 2022). The conjugating enzyme can transfer the ubiquitin from E1 to the substrate and is required for *Notch* signaling activation during *Drosophila* wing development (Gonen et al., 1999; Zhang et al., 2021). Since this gene is reported in the endocytic trafficking of the *Notch* protein, it could potentially influence the endocytic traversion of virus particles in *A. gossypii*. The overexpression of this gene may partially be responsible for the efficient retention and inoculation of CLRDV upon acquisition of the virus from cotton than the other three hosts (Pandey et al., 2022). This gene also is vital for insect defense against pathogens (Lemaitre and Hoffmann, 2007).

The immune system of insects helps them defend against pathogens (Wang et al., 2016). The change in the expression level of the genes related to the immune system and different signaling pathways in *A. gossypii* varied between the host species from which the virus was acquired. The genes related to the MAPK signaling pathway were differentially expressed in *A. gossypii* that acquired the virus from all hosts. In contrast, genes related to the JAK–STAT signaling pathway were only differentially expressed in *A. gossypii* that acquired the virus from okra plants. The JAK–STAT signaling pathway triggers insects’ innate immunity and antiviral responses (Dostert et al., 2005; Hedges and Johnson, 2008; Kingsolver et al., 2013). One of the genes associated with the immune system in aphids is *Cathepsin B* (Kubo et al., 2012; Quan et al., 2019). *Cathepsin B* is an aphid gut cysteine protease that regulates host protein activity and plays a role in the recognition and movement of viruses at the gut level (Pinheiro et al., 2017; Heck and Brault, 2018). *Cathepsin B* gene transcripts were overexpressed in *A. gossypii* acquiring CLRDV from infected cotton and okra plants alone. It was reported previously that the *cathepsin B* expression in aphids depends significantly on the host species (Pinheiro et al., 2017). This may partially explain the identification of the *cathepsin B* gene only in two host species in this study. The overexpression of the *cathepsin B* gene also was reported from *M. persicae* that acquired TuYV from infected plants (Marmonier et al., 2022). In contrast, *M. persicae* that acquired PLRV from infected plants had reduced expression of *cathepsin B*, which was associated with enhanced PLRV transmission (Pinheiro et al., 2017). The overexpression of the *cathepsin B* gene in *A. gossypii* acquiring the virus from okra plants may be one of the reasons for reduced CLRDV retention and subsequent inoculation in the previous study (Pandey et al., 2022). However, it does not explain the CLRDV retention and inoculation results obtained in *A. gossypii* upon acquisition from cotton despite overexpression of *cathepsin B* genes (Pandey et al., 2022).

For successful aphid-mediated transmission of circulative non-propagative phytoviruses such as poleroviruses, the virus capsid protein must interact with putative receptors at the midgut and accessory salivary glands (Gray and Gildow, 2003). One of the critical gene families identified in this study was *serine/threonine kinase receptors*. These genes were differentially expressed in both directions (over and under) in *A. gossypii* upon virus acquisition from four different host species in this study. This gene also was identified as one of the hub genes in the largest module (turquoise) in WGCNA in this study. The differential expression of these genes also was reported in whiteflies that acquired another group of persistent non-propagative circulative phytoviruses (begomoviruses) compared with their non-viruliferous counterparts (Mugerwa et al., 2022). *Serine/threonine kinase*, in mammalian cells, also has been recorded to play a vital role in clathrin-mediated endocytosis of the rabies virus (Wang et al., 2020a). The identification of these receptors in this study highlights their potential role in the circulative movement of poleroviruses in their aphid vectors. However, the role of host plants in the differential expression of these receptors’ genes cannot be explicitly established in this study.

Another important group of DEGs is associated with xenobiotics detoxification. Genes such as *cytochrome P450*, *ATP binding cassette transporters* (*ABC*s), and *UDP-glycosyltransferases* (*UGT*s) were differentially expressed in *A. gossypii* that acquired CLRDV from different host species in this study. These detoxification genes are essential for the adaption of insects to different host plants (Quan et al., 2019). Among them, *cytochrome P450* genes were mainly overexpressed in *A. gossypii* that acquired CLRDV from infected okra and prickly sida plants. However, they were not differentially expressed in *A. gossypii* that acquired CLRDV from other host species. The overexpression of *cytochrome P450* genes also was reported in *M. persicae* upon PLRV acquisition (Patton et al., 2021). Therefore, the overexpression of these genes may assist aphids enhancing the tolerance of non-desirable host plants, which could ultimately help in virus transmission and epidemics (Casteel and Jander, 2013).

### Co-expression networks and hub genes from candidate modules

Gene co-expression networks attained through WGCNA also identified modules of highly correlated genes associated with virus transmission and vector performance. Three of the four most interacting hub genes were annotated: XM_027993669.1 (*trichohyalin*-like), XM_027997289.1 (uncharacterized protein), XM_027997209.1 (*glucose dehydrogenase [FAD*, *quinone*]-like), and XM_027983368.1 (*neuroendocrine convertase 1*-like). A previous study speculated the role of trichohyalin during immune defense via tissue remodeling and interaction with cuticular binding blocks that facilitate encapsulation (Takase and Hirai, 2012; Simons, 2015; Feng et al., 2022). Similarly, the *FAD glucose dehydrogenase* is a detoxification enzyme, the overexpression of which induces defense by reducing quinone in parasite-infected bumble bees (Stone and Yang, 2006; Giacomini et al., 2023). The *neuroendocrine convertase 1*, also called proprotein convertase (*PC1/3*), is a neuropeptide involved in regulating insect growth and development (Greenlee and Harrison, 2004; Callier and Nijhout, 2011). Another earlier study also has identified the essential role of *PC1/3* in maintaining metabolic balance and nutrient-dependent fertility in adult beetles (Fritzsche and Hunnekuhl, 2021). These highly interacting hub genes could play a significant role in the development and defense mechanisms in *A. gossypii* following CLRDV acquisition and could be important targets for future investigation.

## Conclusion

Gene expression profiles varied in substantial magnitude with hosts even within the same family and when interacting with the same virus isolate. Only four common genes were identified between the aphids acquiring the virus from four host species. Several unique genes associated with virus infection, immunity, growth, and development were identified among all DEGs analyzed. In addition, DEG families identified in this study indicate similarity with studies involving other persistent non-propagative viruses (Li et al., 2019, 2020; Patton et al., 2021; Catto et al., 2022; Chesnais et al., 2022; Marmonier et al., 2022; Mugerwa et al., 2022). Despite the same gene families that were identified in aphids from multiple hosts, the directional patterns of these DEGs varied (in some instances overexpressed and in other instances underexpressed) with acquisition hosts. These results reiterate that host plants could have an outsized role in determining vector–virus interaction outcomes. Future studies should examine this phenomenon in other virus pathosystems as well and evaluate the effects of differential gene expression patterns in vectors on their fitness parameters and functionally associate unique gene–fitness as well as gene expression directional pattern–fitness relationships.

## Data availability statement

The data for this article can be found in the NCBI GenBank repository at https://www.ncbi.nlm.nih.gov/ under the BioProject PRJNA934319. Raw sequence data for the BioSamples: SAMN31430961-SAMN31430989 are deposited in the SRA accessions: SRR23579709-SRR23579737.

## Ethics statement

The manuscript presents research on animals that do not require ethical approval for their study.

## Author contributions

SP: Data curation, Formal analysis, Investigation, Methodology, Software, Validation, Writing – original draft, Writing – review & editing. MC: Data curation, Formal analysis, Methodology, Software, Validation, Writing – review & editing. PR: Funding acquisition, Resources, Supervision, Writing – review & editing. SB: Resources, Supervision, Writing – review & editing. AJ: Resources, Supervision, Writing – review & editing. RS: Conceptualization, Funding acquisition, Project administration, Resources, Supervision, Validation, Visualization, Writing – review & editing.

## Supplementary material

Supplementary Table 2 List of DEGs associated with significant GO terms in *A. gossypii* that acquired CLRDV from infected cotton plant.

Supplementary Table 3 List of DEGs associated with significant GO terms in *A. gossypii* that acquired CLRDV from infected hibiscus plant.

Supplementary Table 4 List of DEGs associated with significant GO terms in *A. gossypii* that acquired CLRDV from infected okra plant.

Supplementary Table 5 List of DEGs associated with significant GO terms in *A. gossypii* that acquired CLRDV from infected prickly sida plant.

Supplementary Table 7 DEGs associated with immune pathways in *A. gossypii* that acquired CLRDV from alternate hosts.

Supplementary Table 8 DEGs associated with reproduction in *A. gossypii* that acquired CLRDV from alternate hosts.
