# Supplementary material for: Aphid gene expression following polerovirus acquisition is host species dependent
Source: Front Plant Sci. 2024 Mar 8;15:1341781. doi: 10.3389/fpls.2024.1341781 (PMC10957536; doi:10.3389/fpls.2024.1341781)
Supplement: Supplementary file 1 [file DataSheet_1.pdf]

Aphid gene expression following polerovirus acquisition is host species dependent

Sudeep Pandey[1], Michael Catto[2], Phillip Roberts[3], Sudeep Bag[4], Alana L. Jacobson[5], Rajagopalbabu Srinivasan[1]*

[1]Department of Entomology, University of Georgia, Griffin, GA 30223; USA (S.P., R.S.)

[2]Department of Entomology, University of Georgia, Athens, GA 30602; USA (M.A.C.)

[3]Department of Entomology, University of Georgia, Tifton, GA 31794; USA (P.R.)

[4]Department of Plant Pathology, University of Georgia, Tifton, GA 31794; USA (S.B.)

[5]Department of Entomology and Plant Pathology, Auburn University, AL 36849; USA (A.L.J.)

*** Correspondence:**
Rajagopalbabu Srinivasan

babusri@uga.edu

Figure S1: Summary of RNA sequencing:

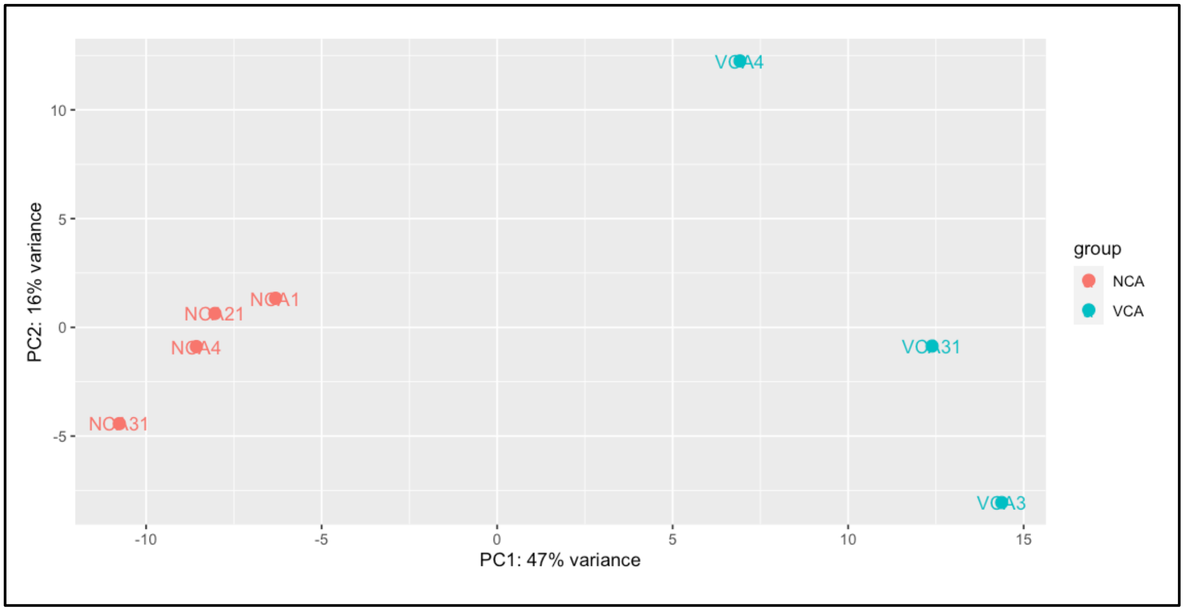

(a)

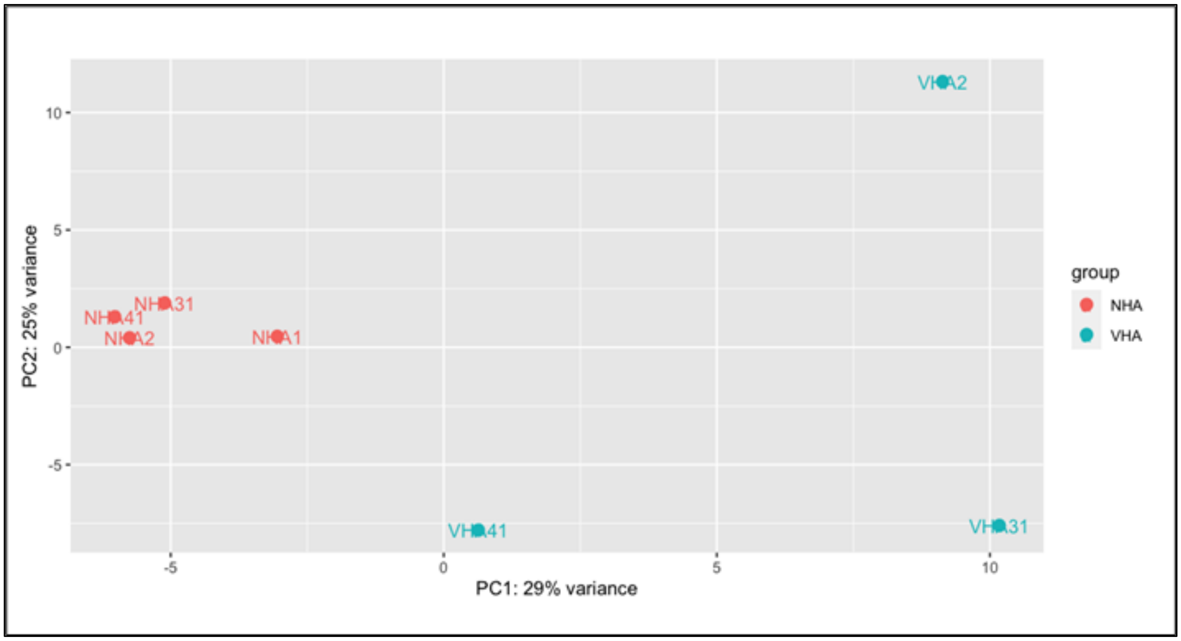

(b)

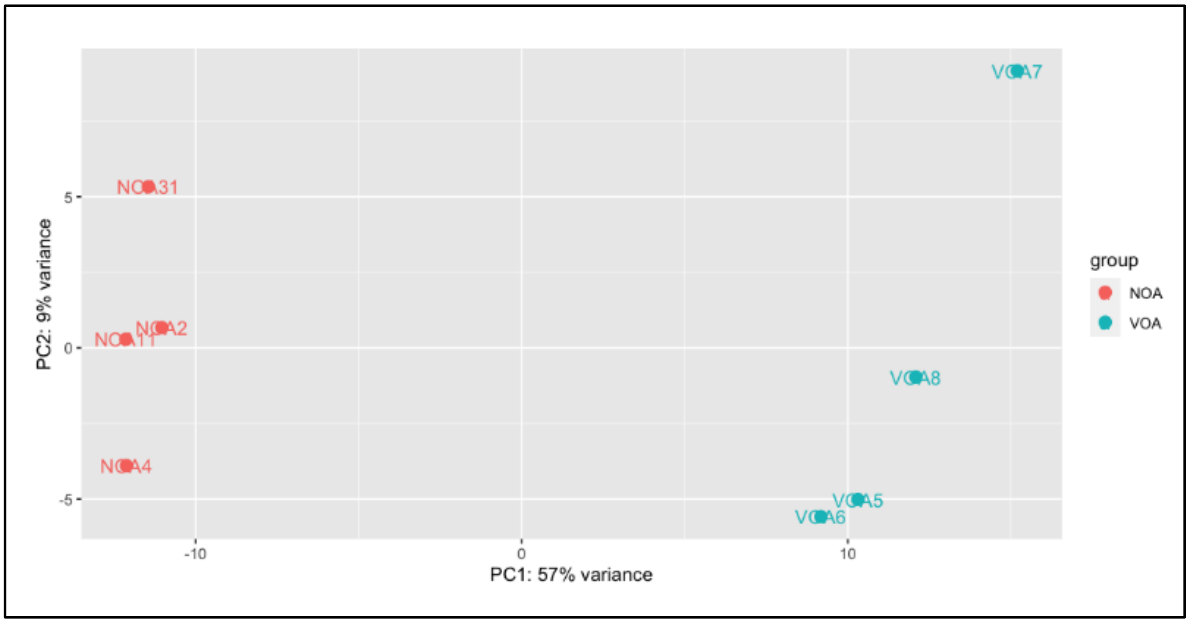

(c)

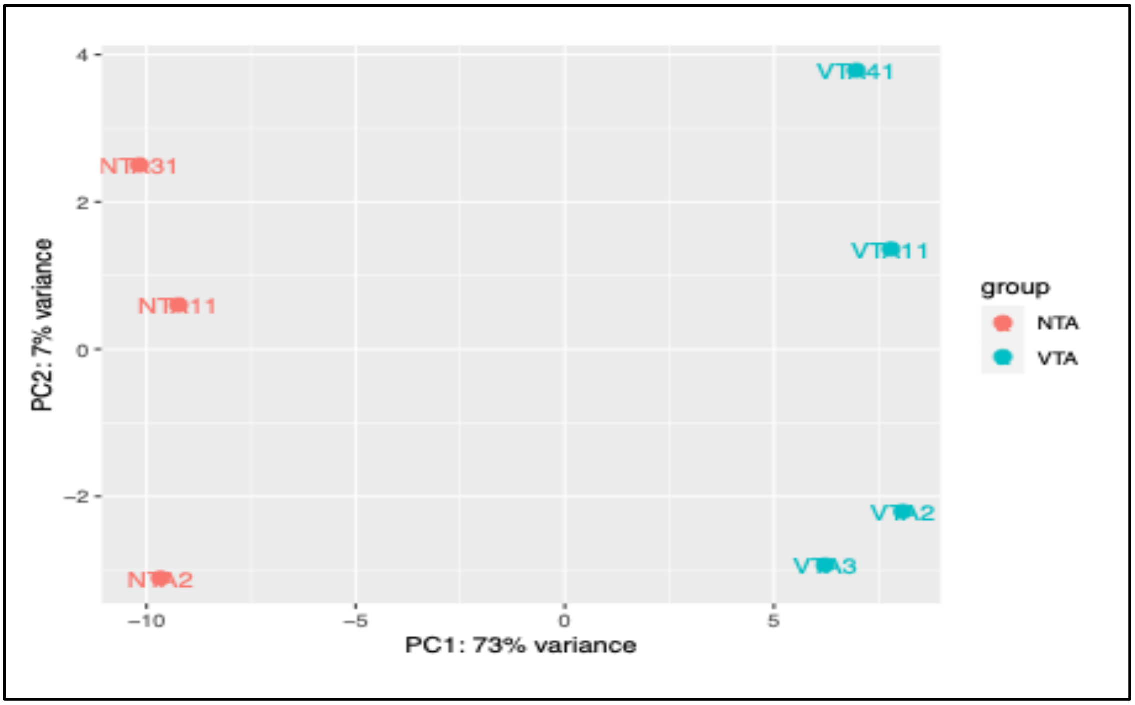

(d)

Figure S1. Principal component analysis based on the gene expression levels in viruliferous *A. gossypii* that acquired CLRDV from infected: (a) cotton, (b) hibiscus, (c) okra, and (d) prickly sida clustered together according to being either viruliferous or non-viruliferous. NCA = non-viruliferous aphid from cotton, VCA = viruliferous aphid from cotton, NHA = non-viruliferous aphid from hibiscus, VHA = viruliferous aphid from hibiscus, NOA = non-viruliferous aphid from okra, VOA = viruliferous aphid from okra, NTA = non-viruliferous aphid from pricklysida, VTA = viruliferous aphid from prickly sida.

Figure S2: Overview of DEGs

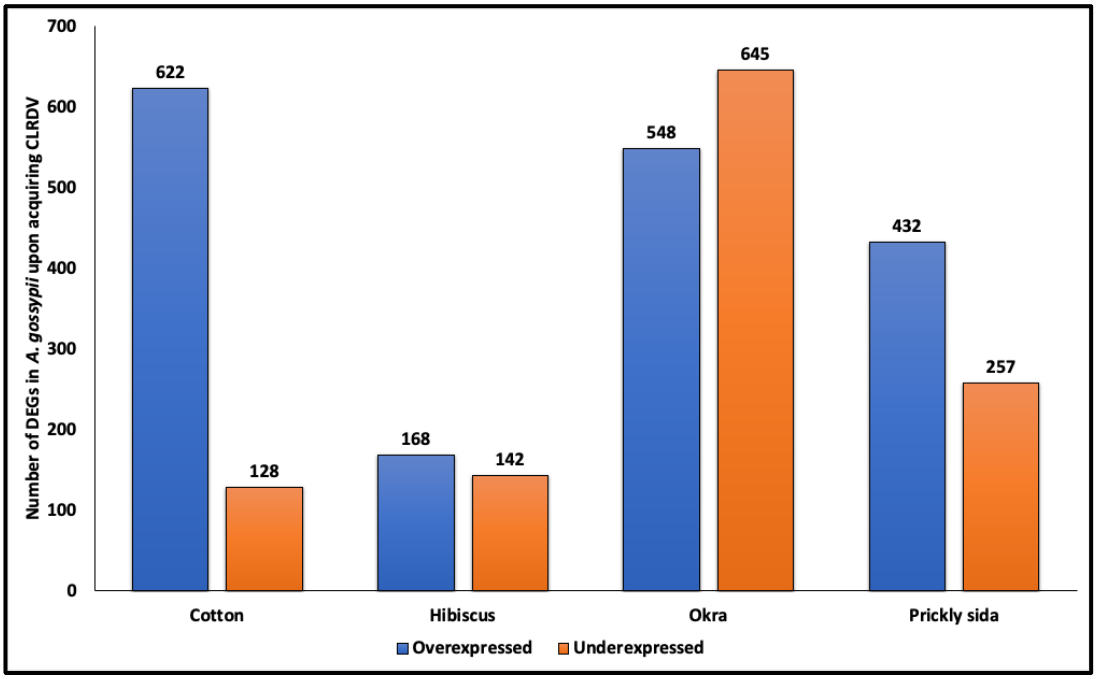

Figure S2. Number of differentially expressed genes (DEGs) in in viruliferous *A. gossypii* adults that acquired CLRDV from infected cotton, hibiscus, okra, and prickly sida plants, compared with non-viruliferous aphids.

Figure S3: Validation of transcriptomics data using RT-qPCR

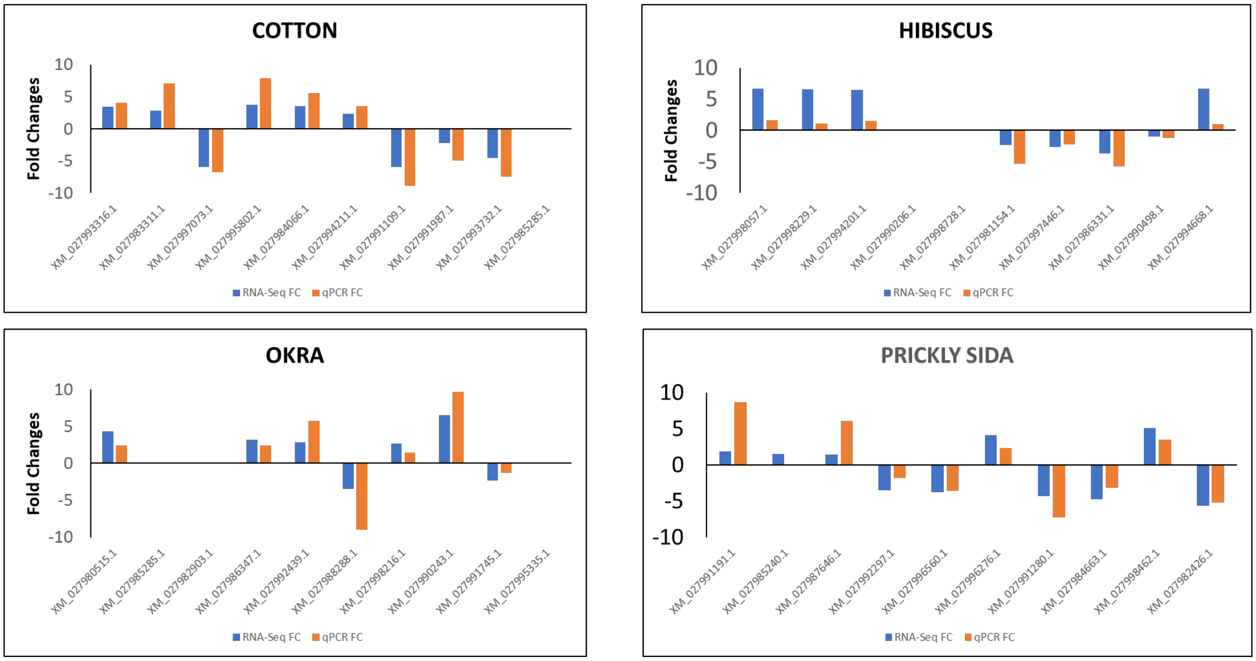

Figure S3: Comparison of expression levels of DEGs in *A. gossypii* adults that acquired CLRDV

from infected cotton, hibiscus, okra, and prickly sida plants analyzed using RNA-seq or RT-qPCR methods.

Figure S4: Functional annotation of DEGs in *A. gossypii* that acquired CLRDV from infected hibiscus plants

A total 174 of the 310 DEGs in *A. gossypii* that acquired CLRDV from infected hibiscus were assigned functional groups under three classification systems: biological process (171 genes), molecular function (158 genes), and cellular component (158 genes). Forty-nine GO terms were assigned under the biological process category, of which only three terms (pigment metabolic process, metabolic process, and cell cycle) were significant (Additional file 1: Figure S3a, Additional file 2: Table S3). Twenty-seven GO terms were assigned under the molecular function category, only two (transferase activity and protein tag) of which were significant (Additional file 1: Figure S3b, Additional file 2: Table: S3). Twenty-two GO terms were identified in the cellular component category; none was significant (Additional file 1: Figure S3c, Additional file 2: Table S3).

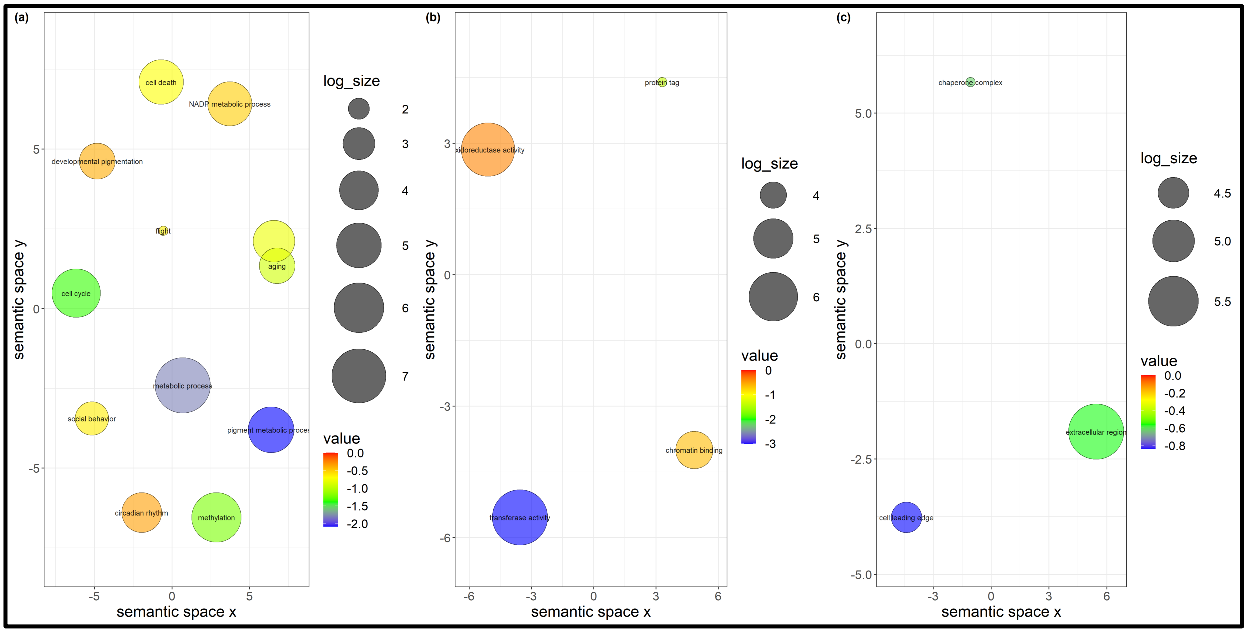

Figure S4. Scatterplots showing (a) biological process, (b) cellular component, and (c) molecular function gene ontology terms for DEGs in viruliferous *A. gossypii* adults that acquired CLRDV from infected hibiscus plant. Cluster representatives in a two-dimensional space were derived by applying multidimensional scaling to a matrix of the semantic similarities of the gene ontology terms. The bubble color indicates the p-value, and the size indicates the frequency of the GO term in the underlying GOA database.

Figure S5: Functional annotation of DEGs in *A. gossypii* that acquired CLRDV from infected okra plants

Overall, 707 of the 1193 DEGs in *A. gossypii* that acquired CLRDV from infected okra were assigned functional groups under three classification systems: biological process (691 genes), molecular function (645 genes), and cellular component (597 genes). Fifty-five GO terms were assigned under the biological process category, of which only three terms (metabolic process, grooming behavior, and aging) were significant (Additional file 1: Figure S4a, Additional file 2:

Table S4). Forty-two GO terms were assigned under the molecular function category, only one (structure molecular activity) was significant (Additional file 1: Figure S4b, Additional file 2: Table S4). Thirty-seven GO terms were identified in the cellular component category, and one of those (extracellular region) was significant (Additional file 1: Figure S5c, Additional file 2: Table S4).

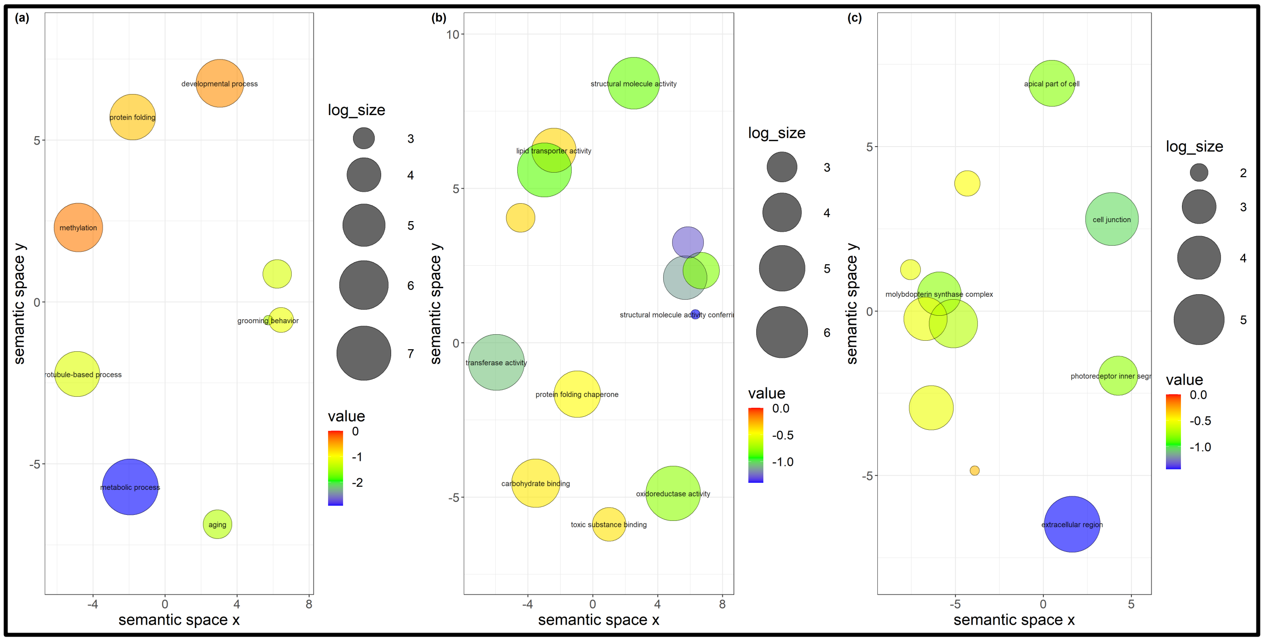

Figure S5. Scatterplots showing (a) biological process, (b) cellular component, and (c) molecular function gene ontology terms for for DEGs in viruliferous *A. gossypii* adults that acquired CLRDV from infected okra plant. Cluster representatives in a two-dimensional space were derived by applying multidimensional scaling to a matrix of the semantic similarities of the gene ontology terms. The bubble color indicates the p-value, and the size indicates the frequency of the GO term in the underlying GOA database.

Figure S6: Functional annotation of DEGs in *A. gossypii* acquiring virus from CLRDV-infected prickly sida plants

Lastly, a total of 371 of the 689 DEGs in *A. gossypii* that acquired CLRDV from infected prickly sida were assigned functional groups under three classification systems: biological process (361 genes), molecular function (334 genes), and cellular component (321 genes). Fifty-two GO terms were assigned under the biological process category, of which only two terms (locomotion and protein folding) were significant (Additional file 1: Figure S5a, Additional file 2: Table S5). Thirty-seven GO terms were assigned under the molecular function category, only three (structure molecule activity, structural constituent of muscle, and transferase activity) of which were significant (Additional file 1: Figure S5b, Additional file 2: Table: S5). Twenty-eight GO terms were identified in the cellular component category, and two (cytochrome complex and basal part of cell) of those were significant (Additional file 1: Figure S5c, Additional file 2: Table S5).

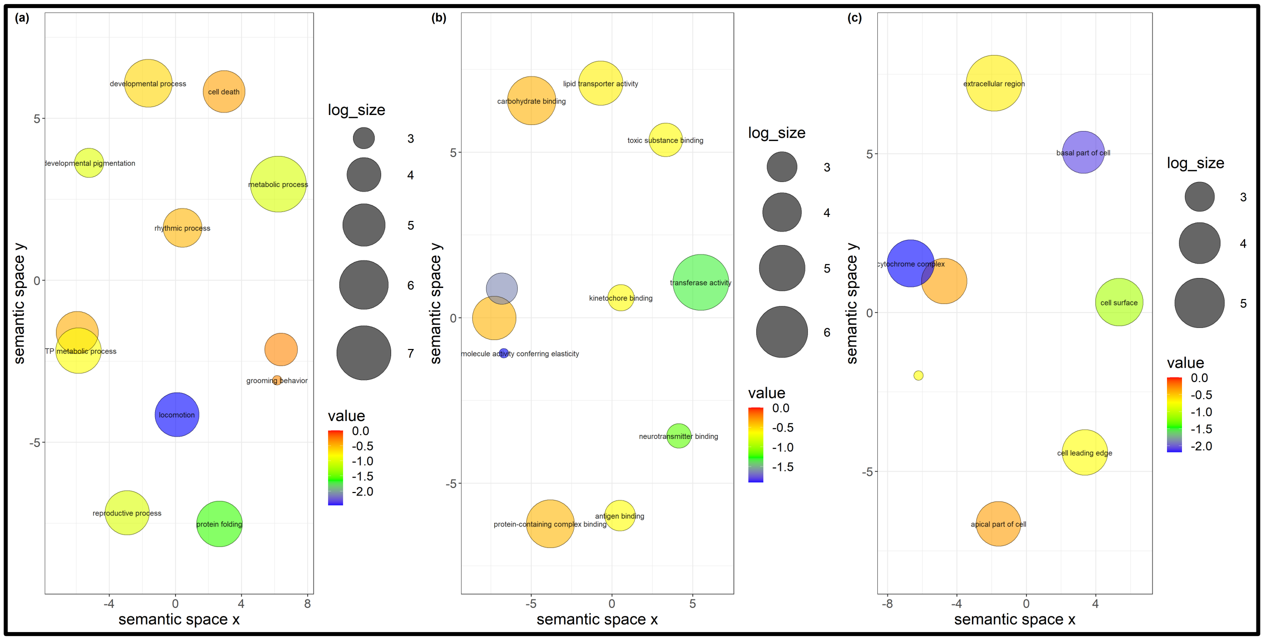

Figure S6. Scatterplots showing (a) biological process, (b) cellular component, and (c) molecular function gene ontology terms for for DEGs in viruliferous *A. gossypii* adults that acquired CLRDV from infected prickly sida plant. Cluster representatives in a two-dimensional space were derived by applying multidimensional scaling to a matrix of the semantic similarities of the gene ontology terms. The bubble color indicates the p-value, and the size indicates the frequency of the GO term in the underlying GOA database.
